# Fire susceptibility assessment in the Carpathians using an interpretable framework

**DOI:** 10.1038/s41598-025-10296-4

**Published:** 2025-08-18

**Authors:** Melinda Manczinger, László Kovács, Tibor Kovács

**Affiliations:** 1 Doctoral School of Economics and Business Informatics, Corvinus University of Budapest, Budapest, 1093 Hungary; 2 Institute of Data Analytics and Information Systems, Department of Statistics, Corvinus University of Budapest, Budapest, 1093 Hungary; 3 Institute of Data Analytics and Information Systems, Department of Information Systems, Corvinus University of Budapest, Budapest, 1093 Hungary

**Keywords:** Climate change, Carpathians, Machine learning, Fire, Interpretability, Feature selection, Environmental sciences, Climate change

## Abstract

Climate change endangers the Carpathian region by increasing the risk of fires. In response, our study provides a harmonised dataset with twenty-seven variables and develops an interpretable machine learning-based framework for assessing fire susceptibility across all seven countries of the region. We applied a two-stage process: first, using various feature selection techniques to refine predictors before the modeling phase, and second, utilising the SHAP framework to interpret model predictions. Between these steps, advanced machine learning models were optimised and trained in the H2O environment, demonstrating high predictive accuracy. Our findings revealed eight fire susceptibility clusters. The resulting dataset, susceptibility maps, and detailed interpretative insights serve as a valuable resource for local communities and policy-makers in the region.

**Supplementary Information:**

The online version contains supplementary material available at 10.1038/s41598-025-10296-4.

## Introduction

Wildfires are increasingly recognised as a major global challenge, becoming more frequent and severe under the pressures of climate change^1–4^. While regions such as California, Australia, and Mediterranean Europe have historically dominated wildfire research, mounting evidence now points to Central and Eastern Europe – including the Carpathians – as a zone of growing vulnerability^5–8^. The unprecedented wildfire season across Europe in 2023 starkly illustrated this shift, with extensive damage reported even in areas previously considered low risk^9^. Historical events such as the severe 2003 drought that impacted sessile oak (*Quercus petraea*) stands in Hungary, and the 2004 windstorm that devastated Norway spruce (*Picea abies*) forests in Slovakia, further underscore the susceptibility of the region to extreme environmental disturbances^10,11^.

Mountainous landscapes like the Carpathians – the largest mountain range in Central and Eastern Europe^12^ – are especially vulnerable. Their steep terrain, fragmented land ownership, and the complex interactions among climatic, ecological, and socio-economic factors amplify both ignition potential and suppression difficulty. Despite the ecological role that natural fires occasionally play in these ecosystems^13^, increasing fire frequency and intensity could drastically reduce essential ecosystem services^14^, threaten biodiversity^15,16^, and undermine the economic stability of sectors such as forestry, agriculture, and tourism^17–19^. Despite these substantial ecological, economic, and social risks, comprehensive fire risk assessments across the Carpathian region remain scarce and are typically limited to isolated, localised studies^7,20,21^.

Machine learning has moved from a niche curiosity to a cornerstone of wildfire research over the past quarter-century^22^. With access to high-quality data, machine learning models can uncover complex, non-linear relationships between environmental and anthropogenic variables, and produce probabilistic maps of fire susceptibility or ignition risk^22^. Successful applications have already been reported in diverse geographic contexts – including China^23,24^, Ecuador^25^, Iran^26,27^, Vietnam^28,29^, the Mediterranean basin^30,31^, Italy^32^, Serbia^33,34^, and Poland^35^ – demonstrating broad applicability. Because no single algorithm universally outperforms others, researchers often explore a variety of techniques and hybrid solutions^29^. A global review of studies from 2001 to 2021 identified 33 different models used in forest fire susceptibility mapping; notably, 73% of these were employed in only one study^36^. The most commonly applied methods included Logistic Regression (statistical), Random Forest (machine learning), and the Analytical Hierarchical Process (knowledge-based), with Random Forest generally achieving the highest average accuracy. While some studies focus on a single model in-depth^23,32,35^, many compare two or more algorithms – often confirming Random Forest’s superior performance^24,30,33,34^. Decision-tree based algorithms are widely used across many disciplines, including fire science, due to their intuitive logic and strong performance in both classification and regression tasks. Although often considered borderline “black box” models, they are valued for their rule-based structure, which segments observations into increasingly homogeneous groups^37^. These splits aim to maximise predictive power, typically assessed metrics like information entropy or the Gini coefficient^38^. Increasingly, broader model-to-model comparisons also include Support Vector Machines, Neural Networks, Naïve Bayes, and Classification and Regression Trees^26–29^. Across this literature, a consensus emerges: wildfire ignition is fundamentally non-linear, and models that capture this complexity yield the best performance. Ensemble and hybrid machine learning approaches generally outperform simpler statistical and knowledge-based methods^36^, while deep learning and hybrid architectures typically deliver the highest predictive accuracy, at the expense of interpretability^39^. Recent studies have further highlighted the increasing importance of integrated remote sensing and deep learning models, emphasizing their applicability beyond wildfire research^40–42^.

Predictive variables in wildfire modelling typically fall into four well-established categories: climatic conditions, vegetation characteristics, topographic factors, and socio-economic or anthropogenic influences^23,43–45^. Climatic drivers such as temperature, precipitation, wind speed, relative humidity, and air pressure influence the amount and accumulation of combustible forest fuels, the moisture content of the vegetation, and the spread and direction of fires^30^. Vegetation characteristics, including forest cover density and health, directly affect forest flammability and fire spread^46^. Topographic parameters such as elevation, slope, aspect, water bodies, and catchment areas determine microclimates, act as natural firebreaks, and affect fuel distribution and moisture content^47,48^. Anthropogenic activities strongly influence fire regimes in Europe with approximately 97% of wildfires being human-induced^49,50^. Common proxies for human activity include population density^51,52^, unemployment rate^30,53^, GDP per capita^24,54^, proximity to infrastructure such as roads, railways, and power lines^55,56^, distance from settlements and croplands^49,57,58^, land use change^59^, and even illegal landfill density^60^. However, the use of varied datasets, inconsistent preprocessing, feature selection strategies, and differing modelling approaches often results in little agreement regarding the most influential predictors for any given region^23,24,61–67^. This underscores the inherent complexity and context-dependency of fire prediction models – and reveals a fundamental challenge: our understanding of how these variables interact within modelling frameworks remains limited^36^.

Despite considerable progress, critical gaps continue to limit the operational utility of current models. First, explicit feature selection – essential for model transparency and performance – is rarely employed in wildfire studies^68,69^. Second, model interpretability is often lacking, which hampers stakeholder confidence^22,70^. Moreover, regional-scale modelling efforts frequently neglect comprehensive cross-border analyses, leading to fragmented and inconsistent risk assessments across politically divided landscapes^69–72^. This further hinders building models that are genuinely transferable across similar geographic contexts.

To address these gaps, our study provides the first comprehensive, interpretable assessment of vegetation fire susceptibility across the seven countries of the Carpathian region. Specifically, we aim to answer the following questions:

1. How accurately can modern interpretable machine learning models predict fire occurrence in this mountain system?
2. Which climatic, vegetation, topographic, and socio-economic factors most strongly influence fire risk across multiple spatial scales?
3. Can we identify high-risk clusters within the Carpathians?

**Fig. 1:**
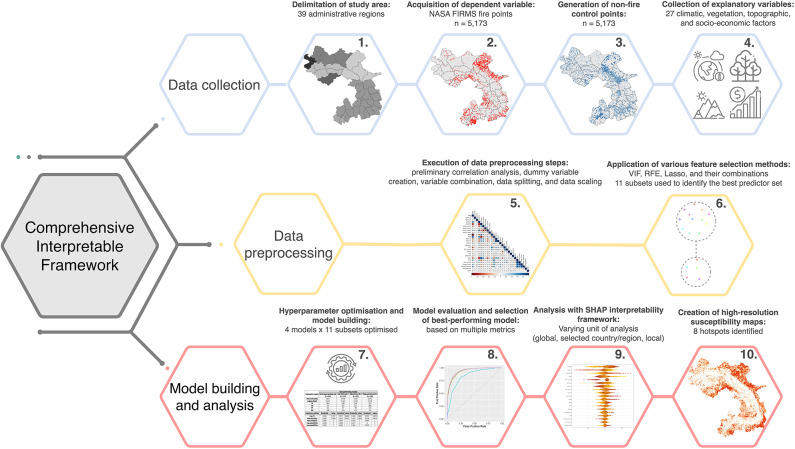
The main steps of our research process.

To answer these questions, we compiled a harmonised dataset comprising twenty-seven variables, primarily derived from satellite remote sensing and interpolated data products. We applied several distinct feature selection methods and their combinations, then optimised multiple advanced machine learning algorithms. We addressed question 1 by rigorously comparing 44 model-feature set combinations. Interpretability was enhanced though the SHapley Additive exPlanations (SHAP)-based attribution analysis, enabling multi-scale insights into the influence of individual predictors. Finally, we addressed question 3 by leveraging H3 hierarchical hexagonal geospatial indexing system. Our analysis revealed that anthropogenic and vegetation-related variables were consistently among the most influential across all regions, while topographic factors exhibited unique patterns warranting further investigation. After identifying the best-performing model, we applied it to generate static susceptibility maps for the entire study area. These maps revealed eight spatial clusters exhibiting elevated fire risk. A summary of our research process is presented in Fig. 1.

## Results

### Fire occurrence in the study region

**Fig. 2:**
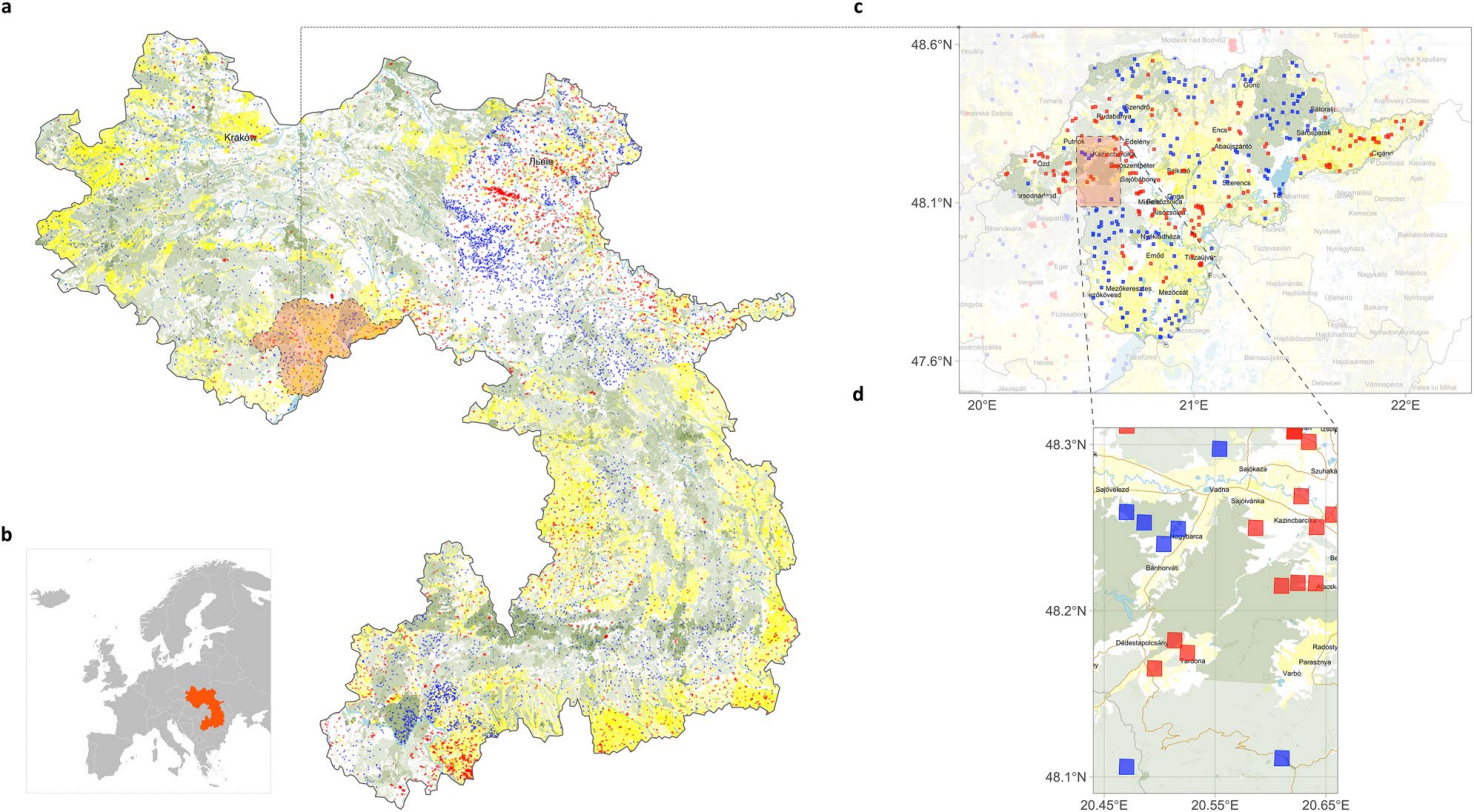
Fire and control points in the Carpathians. Fire points from 2010 to 2020, with ≥ 80% confidence, are shown in red, while negative controls are marked in blue. The landscape is represented using the following OpenStreetMap features: light blue for water bodies (water), green for forested areas (forest), yellow for agricultural lands (farmland), orange for road networks (road), and grey for administrative borders (borders). Cities, towns, and villages are labelled by name. All maps are based on the WGS84 geographic coordinate system. Panels a, c, and d display water, forest, and farmland using the same visual style. **Panel a**: Overview map of the Carpathian region with cities (population > 350,000). **Panel b**: The location of the Carpathians in Europe. **Panel c**: Map of Borsod-Abaúj-Zemplén county (Hungary) displaying towns (population > 1,000), cities (population > 10,000), and borders. **Panel d**: Detailed map of a part of Borsod-Abaúj-Zemplén county including road, towns (population > 1,000), cities (population > 10,000), villages (population > 500), and borders. All maps were created by the authors using R version 4.1.2 (R Core Team, 2021; https://www.R-project.org/) with the packages *rnaturalearth*, *osmdata*, and *ggplot2*.

First, we collected active fire data identified by the Moderate Resolution Imaging Spectroradiometer (MODIS) aboard Terra and Aqua satellites (see Materials). Figure 2, panels a-d provides an overview of the Carpathian Mountains study region, encompassing 39 administrative units distributed across Czechia, Hungary, Poland, Romania, Serbia, Slovakia, and Ukraine (see Materials for county details).

From 2010 to 2020 a total of 5,173 fire incidents were identified, corresponding to a mean of 470 events per year (see Supplementary Table S1). Annual activity peaked in 2012 with 991 fires, while both 2011 (623) and 2015 (581) also exceeded the annual average.

Marked seasonality was evident: fire numbers rose sharply in early spring and reached a maximum in March. Spatial patterns were equally pronounced. Roughly 78% of all fire incidents were concentrated in Ukrainian and Romanian counties, which together occupy the largest share of the mountain chain. Within the range, fires were most frequent at lower elevations along the outer foothills where settlements and agricultural land dominate; high elevation interior ridges experienced comparatively few events (Fig. 2, panel a).

### Feature selection eliminated strongly correlated variables and highlighted the most relevant ones

We analysed twenty-seven candidate predictors spanning four thematic groups – climate, vegetation, topographic, and socio-economic (see Materials for the description of each variable). After running three complementary feature selection families (filter, wrapper, and embedded), ten reduced predictor sets were compared (Table 1). As described in the Methods section, feature selection was performed on 80% and evaluated on 20% of the training set.

Variance Inflation Factor (VIF) screening yielded two predictor subsets (Table 1): the permissive set, which includes 23 predictors with VIF values below 10, and the restrictive set, comprising 20 predictors with VIF values below 5. These two sets differ slightly in the number of variables, but both cover a relatively large number of predictors compared to other subsets in the analysis. When examining multicollinearity in these predictor subsets, we found that Spearman’s correlation coefficients for the permissive set ranged from − 0.754 to 0.916, while the restrictive set had values between − 0.709 and 0.604. The restrictive set substantially lowered maximum pairwise correlation, falling within the typical ± 0.7–0.75 range often used in similar studies to decide upon multicollinear variables^30,33^. This set provided a leaner, less collinear input for subsequent RFE-based selection. Both predictor subsets were fed into RFE as uncorrelated sets.

To boost Recursive Feature Elimination’s (RFE) predictive performance, we used its various adaptations (see Methods). First, we presented the model with correlated (full set of predictors) and uncorrelated (two VIF subsets) predictors. The latter was necessary because collinear variables can distort the assessment of variable importance in Random Forest models^73^. Second, we introduced the concept of the logical rerank argument as there is no consensus in the literature on which approach proves to be more advantageous for our dataset^74,75^. Third, for the correlated set, we also introduced a 1% tolerance level allowing the algorithm some leeway to select a smaller, yet nearly as effective, set of features. As a result, RFE generated six alternative predictor sets (Table 1; Fig. 7). The comparison of the six different RFE sets revealed two key insights: (i) recalculating variable importance before each step had minimal impact on the number of variables included in the resulting subsets, except when the feature set with minimally correlating variables was used as an input; (ii) adjusting the tolerance level when filtering the full feature set led to a smaller subset of variables than starting with already prefiltered, non-correlating variables.

For running the Least Absolute Shrinkage and Selection Operator (Lasso) algorithm, we identified two key $\:\lambda\:$ values, which further determined the predictor subsets used later in the H2O modeling phase (see Methods for details). Using the first, $\:{\lambda\:}_{max}$, led to a subset of 25 predictors, while the second, $\:{\lambda\:}_{1se}$, resulted in a sparser set of 14 variables. Interestingly, as seen in Table 2, this sparser feature set yielded the best performance in the Multiple Logistic Regression (MLR) model.

Comparing all subsets from the different feature selection processes, the **cropland** variable consistently appeared in every subset, indicating its prominence across all methods. Out of twenty-seven variables, feature selection methods identified optimal subsets ranging from 1 to 25 variables. In terms of performance, RFE consistently yielded the best-performing subsets, except for the subset with only one variable. The hybrid combination of RFE and VIF, specifically the RFE-rVIF-F, was the top performer, selecting 17 variables. The best-performing RFE subsets were followed by Lasso, and VIF subsets, respectively. Table 1 shows a summary of the results for each subset. Further evaluation metrics can be found in Supplementary Table S2 and related runtimes in Supplementary Table S3.

**Table 1:**
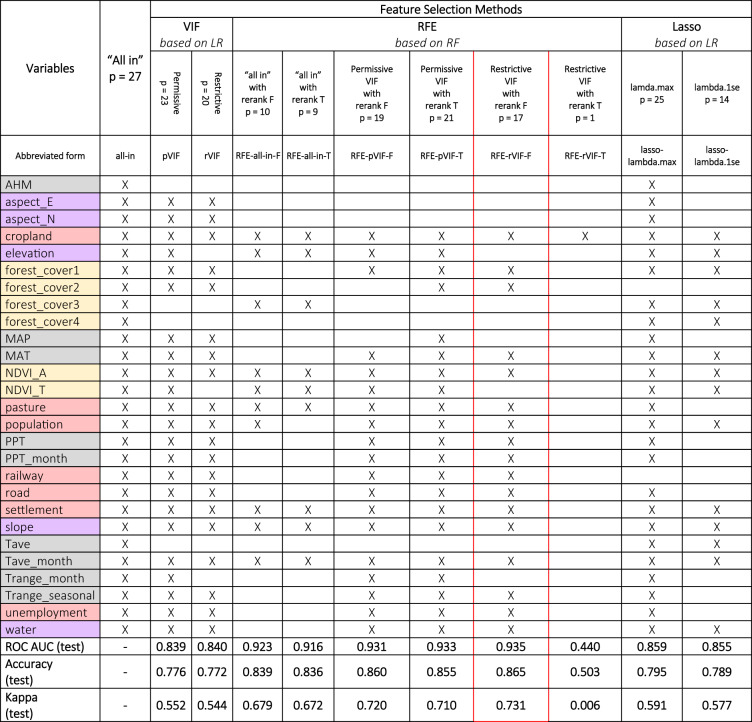
Summary of the feature selection results for each subset. The best-performing subset, based on test ROC AUC, accuracy, and kappa statistics, is highlighted with a red margin. The specific models used for feature selection are indicated in italics under each feature selection method (LR stands for logistic regression and RF for random Forest). We colour-coded features to represent the following categories: red for anthropogenic, orange for vegetation-related, purple for topographic, and grey for climatic factors. These colours are used consistently to represent the analysed variables throughout the study.

### Machine learning models accurately predicted fire occurrence

We selected three tree ensemble models – Distributed Random Forest (DRF), Gradient Boosting Machine (GBM), and eXtreme Gradient Boosting (XGBoost) – due to their popularity and efficiency (see Introduction). Additionally, we included Multiple Logistic Regression (MLR) as a benchmark statistical linear model. We trained the models on 80% of the entire dataset and evaluated on the remaining 20% (see Methods). We used every variable subset generated during feature selection – 10 subsets plus the “all-in” set – for every model type and carried out parameter optimisation for each case separately (see Methods). First, we identified the 44 best-performing models (11 feature sets x 4 models). Next, we selected the best of these models and used it for fire probability calculation. We assessed model performance using the cross-validated ROC AUC metric, which ranges between 0 and 1, 0.5 being a random categorisation and 1.0 being a perfect classification of fire and non-fire points.

**Fig. 3:**
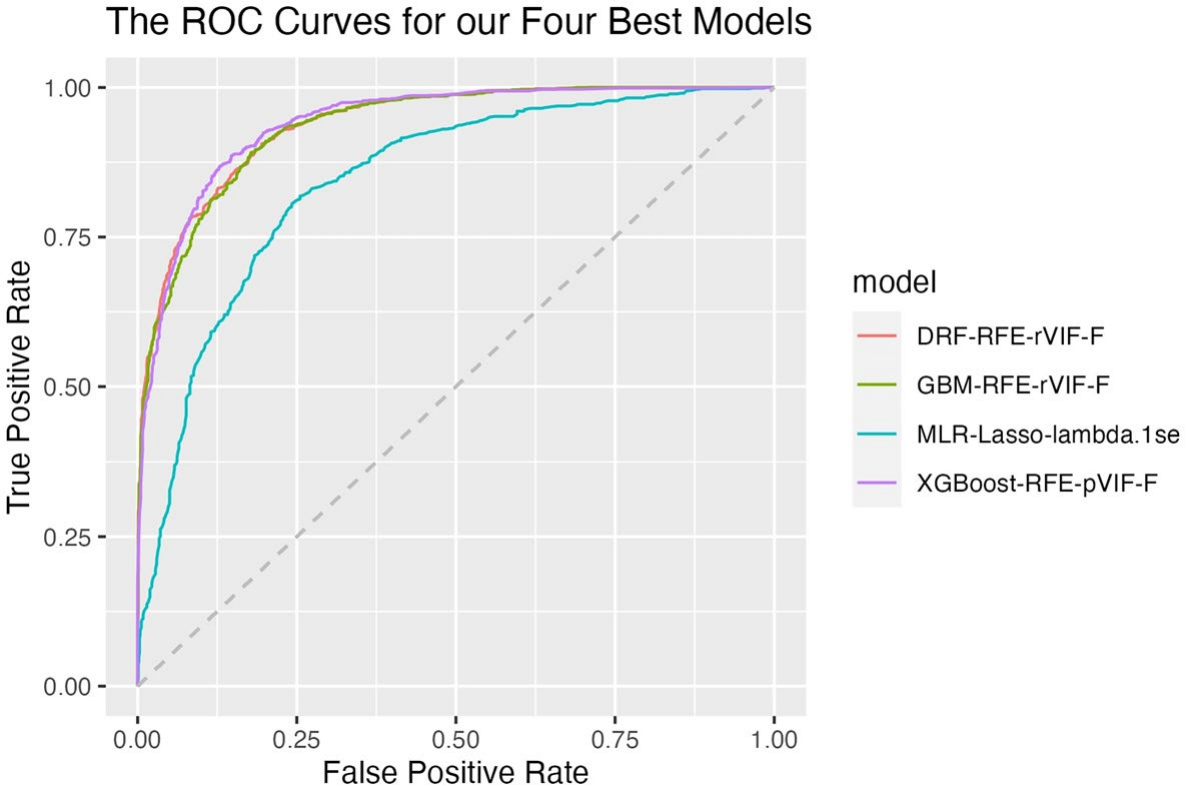
ROC curves for the top four model-subset combinations, with the FP rate on the x-axis and the TP rate on the y-axis.

We present our comprehensive results in Supplementary Tables S4-S7, showcasing the best results of each model-subset combination. Among the models, MLR demonstrated the weakest performance, with test AUC values ranging from 0.791 to 0.857 (see Supplementary Table S4). In contrast, tree-based models generally achieved better test AUC values. The DRF model, for instance, showed AUC values ranging from 0.652 to 0.940 (Supplementary Table S5), with its best performance using the RFE-rVIF-F feature subset (*p* = 17). Similarly, the GBM model had AUC values ranging from 0.662 to 0.937 (Supplementary Table S6), performing best with the same RFE-rVIF-F subset. Although both the DRF-RFE-rVIF-F and GBM-RFE-rVIF-F models delivered slightly lower but still highly accurate results compared to XGBoost-RFE-pVIF-F (see Table 2; Fig. 3), they excelled in maximising the correct identification of positive fire cases (TP) while minimising false positives (FP). These models are particularly well-suited for scenarios where accurate fire prediction is crucial, such as during peak fire seasons when rapid response is essential. Misallocating resources to areas incorrectly identified as high-risk can have serious consequences, including delayed responses to actual fire outbreaks. Therefore, in ecosystems prone to large megafires, such as those in California, we would recommend using one of these algorithms.

The XGBoost models showed AUC values ranging from 0.616 to 0.941 (Supplementary Table S7), with its best performance using the RFE-pVIF-F feature subset with 19 variables. The XGBoost model excelled in maximising the true negative rate (TN) while minimising the false negatives (FN). Given its performance in differentiating true and false negative fires, this model is well-suited for situations where accurately identifying non-fire areas helps in efficient resource allocation. Considering its performance results - achieving the highest F1 and accuracy scores - and its suitability for avoiding undetected fires, we selected the XGBoost-RFE-pVIF-F model with 19 variables as the best-performing model. This model was further chosen for both SHAP interpretability analysis and susceptibility assessment. Notably, our findings suggest that the optimal feature subset comprises around 17–19 features (see Table 2).

**Table 2:** Model evaluation metrics reported on the test dataset for the best-performing models determined by the highest ROC AUC values. True positives (TP), true negatives (TN), false positives (FP), and false negatives (FN) are reported on the F1-optimal threshold, while maximal evaluation metrics are reported at their optimal thresholds.

Evaluation metric	Best-performing models							
	MLR-Lasso-lambda.1se (*p* = 14)		DRF-RFE-rVIF-F (*p* = 17)		GBM-RFE-rVIF-F (*p* = 17)		XGBoost-RFE-pVIF-F (*p* = 19)	
ROC AUC (test)	0.857		0.940		0.937		0.941	
Logloss (test)	0.475		0.363		0.323		0.378	
TP	884		948		946		919	
TN	737		821		821		880	
FP	151		87		89		116	
FN	298		214		214		155	
**Maximum metrics**	**Threshold**	**Value**	**Threshold**	**Value**	**Threshold**	**Value**	**Threshold**	**Value**
Max F1	0.435	0.797	0.472	0.863	0.402	0.862	0.452	0.872
Max accuracy	0.609	0.788	0.516	0.857	0.497	0.854	0.558	0.870
Max precision	0.985	1	0.982	1	0.993	1	0.999	1
Max sensitivity	0.018	1	0.108	1	0.027	1	0	1
Max specificity	0.985	1	0.982	1	0.993	1	0.999	1

### SHAP analysis identified the most important global and regional factors influencing fire susceptibility

**Fig. 4:**
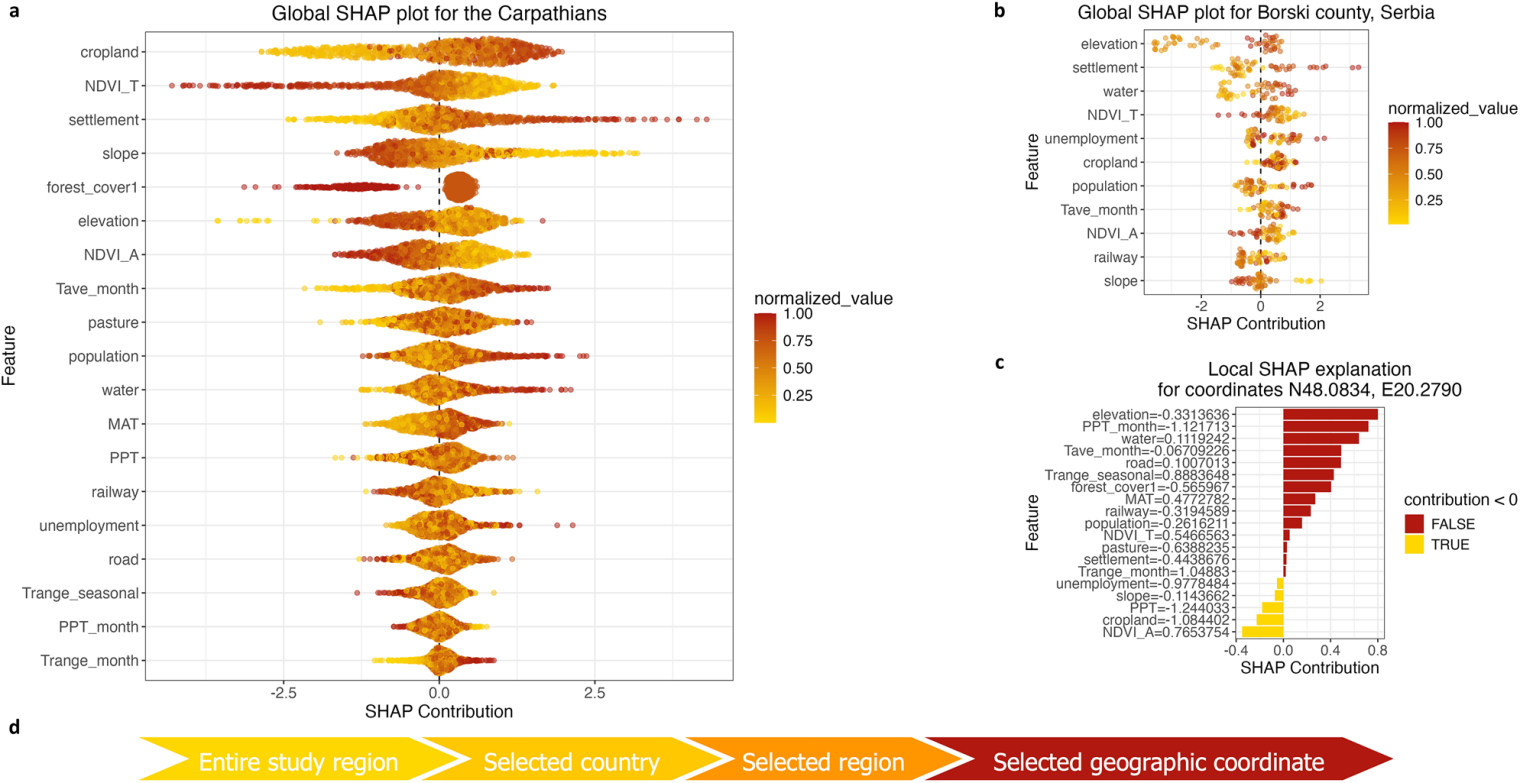
Global and local SHAP plots, providing a detailed visualisation of how features contribute to the model’s predictions. The global plots rank variables by importance based on the average of their absolute SHAP values (y-axis) and show the impact of each feature on the outcome (x-axis) through individual SHAP contribution values for each data point. Feature values are normalised, with red dots representing high values and yellow dots representing low values. SHAP contributions greater than 0 indicate susceptibility to fire, while values below 0 indicate a negative association. **Panel a**: Global SHAP Summary Plot for the entire study region using the XGBoost-RFE-pVIF-F model. **Panel b**: Global SHAP Summary Plot for Borski county, Serbia, using the same model, showing the top 11 variables. **Panel c**: Local SHAP plot showing also scaled feature values on the y-axis for the geographic coordinates N48.0834, E20.2790, near Bekölce, Heves county, Hungary. **Panel d**: Granularity of the SHAP analysis used in the current study.

Using the XGBoost-RFE-pVIF-F model, we calculated SHAP contribution values for both the entire region and specific local areas, as shown in Fig. 4. This approach allowed us to identify the features influencing fire patterns across the entire Carpathian region (panel a), within an individual county (panel b), and at specific coordinates (panel c).

The model’s predictions were significantly influenced by the **cropland** variable, which ranked highest on the y-axis, indicating its status as the most important factor globally. There was a strong correlation between increasing cropland density and the occurrence of fires (see Fig. 4, panel a). This may be attributed to the fact that agricultural lands – closely associated with human activity – often host a high density of fire-prone vegetation, thereby contributing to more frequent fires^76^. However, land cover differences in fire proneness warrants further in-depth investigation, similarly to the Mediterranean Europe^77^. The influence of human activity was further supported by the observed association between fire occurrence and the presence of **pastures** or grasslands. Specifically, regions with particularly low pasture density tended to be more protected from wildfires, while areas with higher densities had greater exposure to fires, which is in line with previous findings in the Mediterranean region^30^. However, this trend was less pronounced for pastures than for croplands, as some grazed areas might have been less exposed to fires due to their reduced combustible materials^56^.

Another example of anthropogenic influence on fires was the effect of **slope** on fire susceptibility. The model shows that steeper slopes, as opposed to flat terrain, were associated with greater protection from fires. This outcome may be linked to the easier human access to flat regions^35^ or the quicker spread of fires in these areas, although opinions on the latter vary^34,77,78^. The case of **population** density and distance from settlements is especially interesting due to the seemingly paradoxical relationship. Our SHAP analysis indicated that high human concentration in a given area was associated with increased fire probability. This is not surprising, given that humans are considered the primary cause of most fires in this ecoregion^55^. Similarly, one might expect that a larger distance from **settlements** would protect them from fires due to decreased human presence. However, our findings suggest the opposite relationship. This counterintuitive result can be attributed to the low fuel content near settlements, as areas near larger cities and towns are less likely to be covered with contiguous vegetation^23^. Additionally, the low distance between **railway** networks and fires reinforced the human influence on fire probability. Although higher **unemployment** rate had a marginal positive effect on fire occurrence in the global model predictions, this trend may be more pronounced in specific regions. Notably, we found no clear global association between **road** networks and fire susceptibility.

High temperatures and low precipitation can facilitate fire ignition^56^, a finding confirmed by our results as well. Mean annual temperature (**MAT**) and monthly mean temperature (**Tave_month**) were positively associated with fire occurrence. Increasing temperatures can lead to higher evaporation rates and drier vegetation, both of which contribute to the likelihood of fires^23,24^. **Trange_month** - the difference between a month’s max and min temperatures – barely influenced the model but the trend indicates that higher values may still stress vegetation and raise fire risk. **Trange_seasonal**, the difference over a season, surprisingly linked to lower fire risk, a result that merits further study. Regarding precipitation parameters, increased monthly precipitation (**PPT_month**) was associated with a marginal negative effect on fire susceptibility, while seasonal precipitation (**PPT**) had an intermediate effect without a clear global pattern. The former effect may arise because higher precipitation increases relative humidity and moistens combustible materials, thereby reducing fire risk^23^.

Climatic factors can significantly influence Normalized Difference Vegetation Index (**NDVI**) values, which reflect the overall health of vegetation^79,80^. Our results demonstrated a strong, consistent negative relationship between NDVI variables (measured from the Terra and Aqua satellites) and fire occurrence. Higher NDVI values, indicating healthier and more robust vegetation, were associated with a lower likelihood of fires. Therefore, lower NDVI values are linked to higher fire susceptibility in two ways: diminished vegetation can raise the risk of fire, while repeated fires can, in turn, drive NDVI values down^80^. Vegetation health is potentially influenced by the proximity of **water** bodies. Indeed, our results indicated that areas distant from surface water resources are associated with higher fire susceptibility, possibly due to the decreased soil moisture content^43^.

Finally, forest coverage – a categorical variable representing vegetation conditions based on canopy density (see Materials) – showed that areas with dense forest cover (**forest_cover1**) are less prone to fires compared to other categories. This is consistent with findings from Southern France reported by^53^. In the Carpathian region, densely forested areas are typically located at higher altitudes (see e.g., Fig. 2, panel a). Consequently, our results indicated that areas at lower **elevations** tend to be more prone to fires, which is in line with previous reports^24,56^.

Fire behaviour can vary significantly across different regions due to local factors such as climate, vegetation composition, topography, and human involvement. One advantage of SHAP is its versatility across various scales, allowing it to be applied to entire study regions, individual counties, and even specific coordinates (see Fig. 4, panel d). This helps to uncover spatial differences and identify patterns. For example, all variables align perfectly with the previous trends in Borski county in Serbia except for the elevation variable, which showed the opposite trend: higher altitudes were associated with increased fire risk (see Fig. 4, panel b). Further investigations are needed to uncover the causes of this trend.

The local SHAP plot reveals which features are most influential for a specific prediction at given coordinates. For example, the local SHAP plot in Fig. 4, panel c explains the model’s prediction of a vegetation fire near Bekölce, Heves county, Hungary, on April 22, 2020. This indicates that high NDVI_A values (healthy vegetation) and low cropland density and PPT values decreased the likelihood of the event being classified as a fire. In contrast, lower elevation, decreased precipitation, and a higher distance from water surfaces had positive contributions, tipping prediction outcome to the fire class. Local media also reported on this fire event, describing a very dry spring period at that time which is reflected in the relationship between our variables. It took a minimum of six hours to extinguish the fire, which resulted in approximately 60 ha of burnt area^81^.

**Fig. 5:**
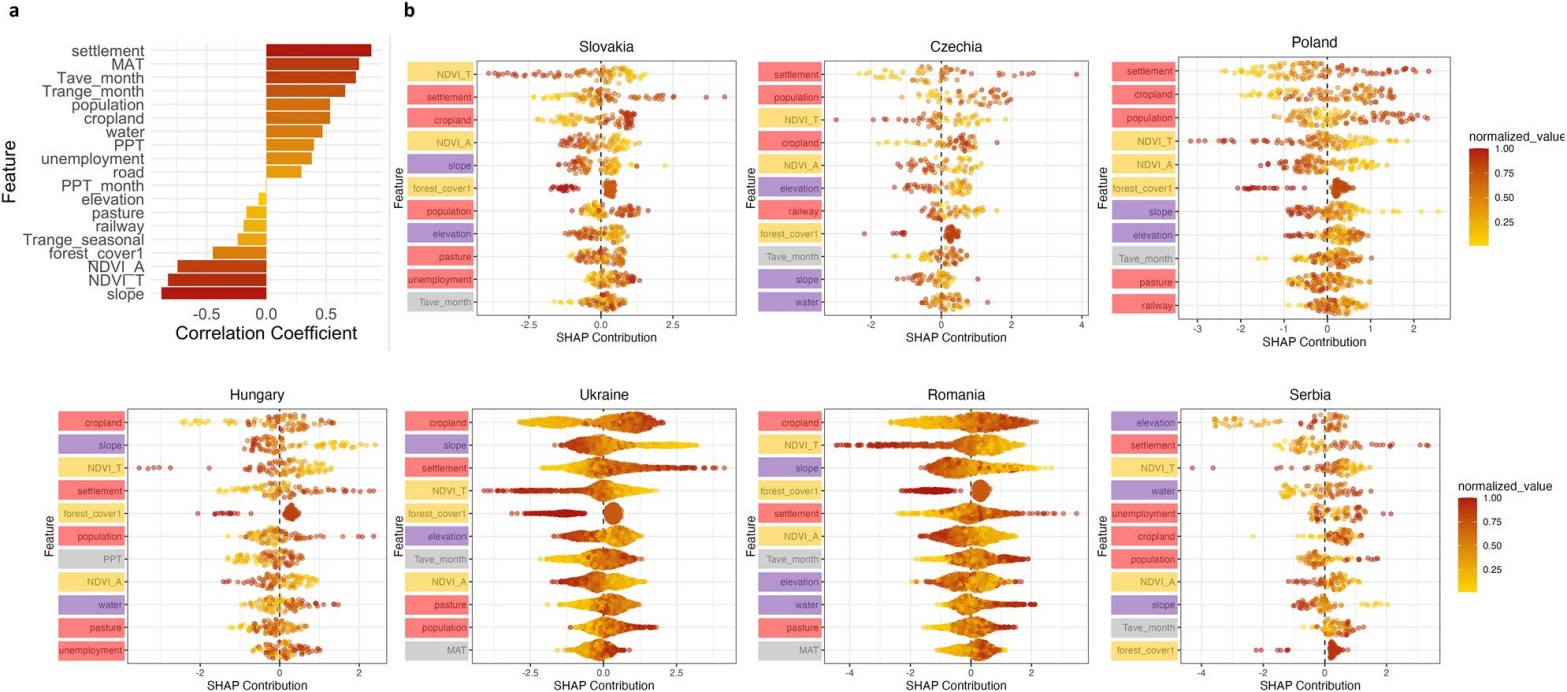
Global SHAP Summary Plots for the seven Carpathian countries. **Panel a**: Bar chart showing Spearman’s correlation coefficients between the actual (scaled) feature values and their corresponding SHAP contributions, calculated on the overall test fire observations (*n* = 1,035). The values of features with higher absolute correlation coefficients are likely to have monotonic relationship with the model’s output. **Panel b**: Global SHAP Summary Plots for each country, with features colour-coded as in Table 1.

To gain deeper insights into the local effects of the examined features on fire susceptibility, we carried out SHAP analysis on a regional basis (see Supplementary Figure S1). The results were then aggregated at the country level to enhance the interpretative power of the SHAP analysis, see Fig. 5. We also sought to identify consistent, monotonic relationships between features and model outputs across countries. To achieve this, we calculated Spearman’s correlation coefficient between the scaled values of each variable and their corresponding SHAP values at each point.

Our SHAP analysis identified the most important variables in each country and showed that anthropogenic factors consistently play a major role in model predictions across all areas (see Fig. 5, panel b). In every country, three to five of the top ten variables were associated with various forms of human activities and showed consistent trends across countries (red background colour on Fig. 5, panel b). The results at the county-scale, shown in Supplementary Figure S1, followed a similar pattern, except for Gorj and Prahova in the Wallachia region of the Southern Carpathians, Romania, where these factors were less influential. Positive correlation coefficients for anthropogenic factors such as proximity to settlements also reinforce their significant impact on the model’s predictions (Fig. 5, panel a).

Climatic, vegetation, and topographic features also played significant roles among the remaining variables. The role of climatic predictors (grey background colour on Fig. 5, panel b) was evident in all the countries, with rising temperatures contributing to increased fire probabilities, particularly in Ukraine and Romania (see also MAT’s and Tave_month’s high positive correlation coefficients on Fig. 5, panel a). Vegetation-related predictors (orange background colour on Fig. 5, panel b) were prominent across all countries, with Slovakia standing out, where NDVI_T emerged as the most important predictor. As already expected from the global results, NDVI exhibited negative correlation with SHAP contributions (Fig. 5, panel a) indicating lower fire probability in case of healthy vegetation.

Finally, topographic factors (purple background colour on Fig. 5, panel b) exhibited an intriguing pattern. These factors were notably more influential in the southeastern side of the Carpathians, particularly in Serbia, Romania, Ukraine, and Hungary, compared to the other countries. Elevation, in particular, stood out as a key predictor in Serbia, emphasizing the role of landscape features in fire dynamics. Proximity to surface water also proved important, particularly in Serbia, Romania, and Hungary, alongside other topographic variables. The indirect effect of the water variable could seem significant as fires are more likely to start where there is more dried combustible material.

### Widespread fire susceptibility identified in the Carpathian region

**Fig. 6:**
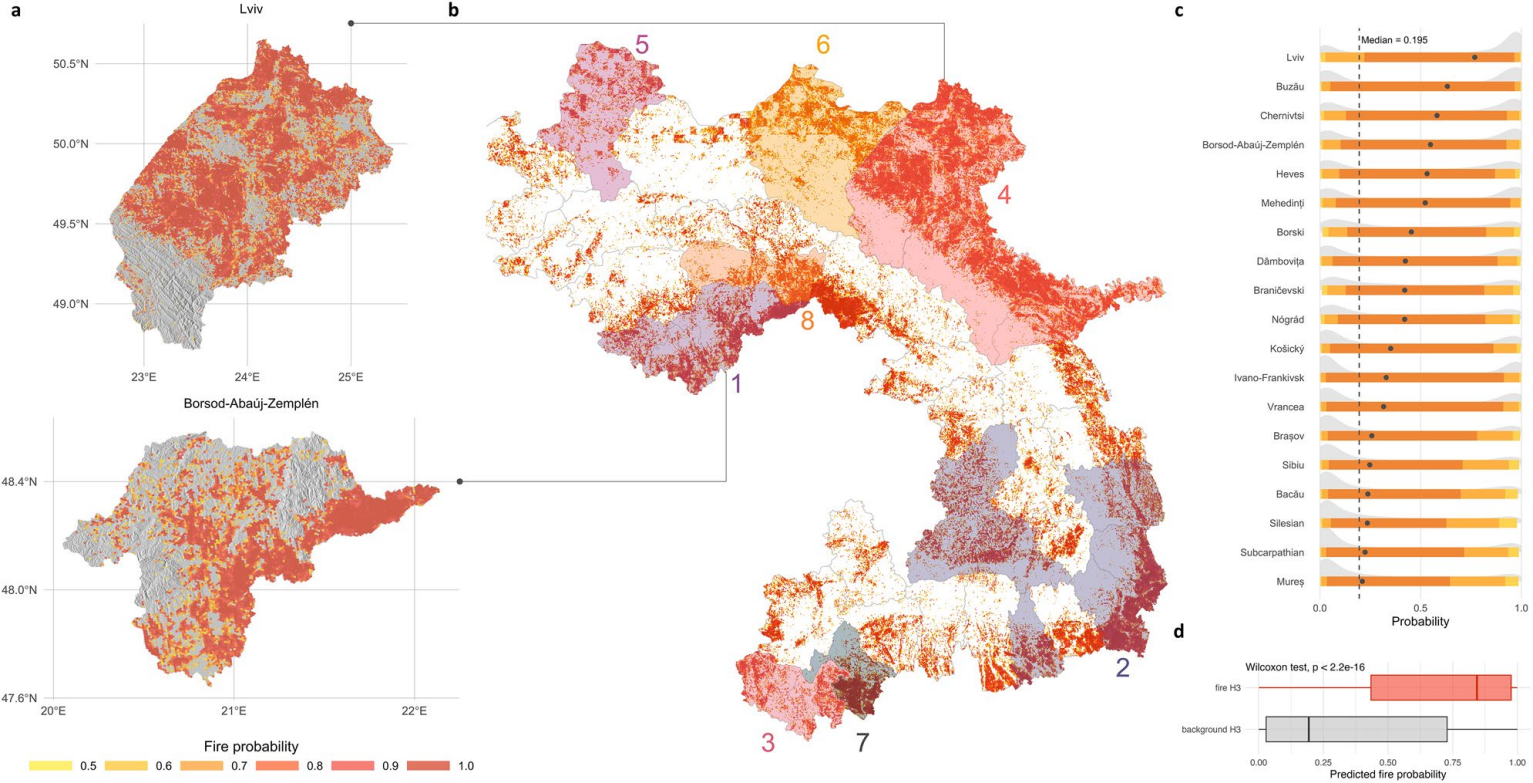
Fire susceptibility in the Carpathian region, in WGS84 projection. **Panel a**: Susceptibility maps showing regions of Lviv (Ukraine) and Borsod-Abaúj-Zemplén (Hungary), highlighting areas with fire probabilities of 0.5 or higher. A grey hillshade was calculated and visualised beneath the fire probability layer for both regions for visualisation purposes. **Panel b**: Susceptibility map of the entire study region utilising 386,885 H3 hexagons. A fire probability threshold of 0.5 was applied for visualisation purposes. Grey lines represent county borders, while coloured numbers indicate identified clusters from spatial clustering. **Panel c**: Ridge plot showing the distribution of fire probabilities by administrative region, arranged in descending order of median fire probability. Only counties with a median fire probability exceeding the overall median (0.195) are shown. Grey-coloured histograms represent the distribution of probabilities for each county, while the dots mark the individual median fire probabilities. The 0.5, 0.8, and 0.95 quantile intervals are also shown, indicating the range where the respective percentages of fire probabilities fall. Plot for all regions can be found in Supplementary Figure S2. **Panel d**: The boxplot indicates the predicted fire probability of hexagons with (*n* = 1,235) and without (*n* = 385,764) fire event detected between 2021 and 2025. All maps were created by the authors using R version 4.1.2 (R Core Team, 2021; https://www.R-project.org/) with the packages *h3jsr* and *ggplot2*.

We assessed fire susceptibility across the Carpathian region using the best-performing XGBoost model (XGBoost-RFE-pVIF-F). Predictions were generated at a 0.74 km^2^ resolution, resulting in a spatial grid of 386,885 hexagonal cells covering a total area of 286,295 km^2^ (see Fig. 6, panel b). Approximately 34% of these cells (133,123 hexagons, equivalent to 98,511 km^2^) exhibited a predicted fire probability of 0.5 or greater.

The predicted fire susceptibility was notably higher in lower elevations and the foothills of the Carpathians. According to the SHAP interpretability analysis, these areas are characterised by more combustible vegetation and conditions conductive to increased fire risk, compared to the typically forested or sparsely vegetated higher elevations. Nonetheless, isolated areas of heightened fire susceptibility were also detected in certain mountainous locations.

To facilitate actionable insights at the administrative scale relevant for fire management^82^, median hexagon-level probabilities ($\:{\stackrel{\sim}{P}}_{c}$) were calculated for each county (NUTS-2 and 3) (see Fig. 6, panel c and Supplementary Figure S2 for all counties). The overall median probability across all counties was 0.195. Counties surpassing this threshold (*n* = 19) were considered as having elevated fire susceptibility (see Fig. 6, panel c). We employed a spatial clustering approach to rank and group these counties based on contiguity within national borders.

Specifically, we employed a queen-contiguity neighbour analysis. We converted the resulting adjacency list to an undirected graph to detect connected regions. To delineate clusters at the national level, we removed links between counties located in different countries. Counties without any neighbours were treated as separate clusters. This method identified four multi-county clusters and four single-county clusters (see Fig. 6, panel b with each cluster numbered and visualised). Notably, countries without major contiguous clusters, such as Czechia, contained sub-regional areas warranting closer attention for fire risk management (see Discussion).

Below we briefly characterise each identified cluster. Median county-level fire susceptibility values ($\:{\stackrel{\sim}{P}}_{c}$) are indicated in brackets.

- **Cluster 1 – Hungarian Carpathians**: Nógrád (0.42), Heves (0.53), and Borsod-Abaúj-Zemplén (0.55) counties constitute Hungary’s cluster. Borsod-Abaúj-Zemplén, the most vulnerable Hungarian county, displays elevated fire probabilities notably around Bükk National Park to the west, Aggtelek National Park to the north, and the Zemplén Landscape Protection Area to the northeast (see Fig. 6, panel a).
- **Clusters 2 and 7 – Romanian Carpathians**: Romania contains two distinct clusters. The first extends from the southeastern outer arc into the inner Transylvanian Plateau and includes Mureș (0.21), Bacău (0.24), Sibiu (0.25), Brașov (0.26), Vrancea (0.32), Dâmbovița (0.42), and Buzău (0.63). Notably, Buzău ranks as the second most fire susceptible county in the entire Carpathian region. The second Romanian cluster consists solely of Mehedinți (0.52), which, despite lacking any adjacent high-risk neighbour within Romania, forms a cross-border cluster with Serbia’s Cluster 3 across the Danube River.
- **Cluster 3 – Serbian Carpathians**: Braničevski (0.42) and Borski (0.45) counties form a contiguous high-susceptibility area along the Danube gorge. This region overlaps Đerdap National Park in Serbia and borders the Iron Gates Natural Park in Romania, highlighting the cross-border nature of the elevated fire risk.
- **Cluster 4 – Ukrainian Carpathians**: Ivano-Frankivsk (0.33), Chernivtsi (0.58), and Lviv (0.77) form Ukraine’s cluster, with Lviv showing the highest median fire susceptibility across the Carpathian region (see Fig. 6, panel a). While Zakarpattia county remains below the overall median susceptibility threshold (see Supplementary Figure S2), a developing elevated susceptibility area seems to be emerging near its border with the Pannonian Basin.
- **Clusters 5 and 6 – Polish Carpathians**: In Poland, the Subcarpathian (0.22) and Silesian (0.24) regions formed two distinct clusters, separated by Lesser Poland region. Subcarpathian borders Cluster 4 in Ukraine. Elevated fire probabilities were primarily concentrated in the northern outer areas, with smaller, sporadic pockets also present on the inner slopes – particularly near Gorczański and Tatrzański National Parks.
- **Cluster 8 – Slovakian Carpathians**: Kosický county (0.35) is the only region in Slovakia with a median fire susceptibility above the overall threshold, with elevated values mainly observed in its lowland areas. This cluster can be seen as a continuation of Hungary’s Cluster 1, as it directly borders the Hungarian counties in that group^83^.

Taken together, the spatial arrangement of clusters highlights *three* prominent cross-border convergence zones: (i) Hungary’s Cluster 1 and Slovakia’s Cluster 8, (ii) Serbia’s Cluster 3 and Romania’s Cluster 7, and (iii) Ukraine’s Cluster 4 and Poland’s Cluster 6. These zones form nearly continuous corridors of elevated fire susceptibility spanning national boundaries, emphasizing that fire risk is inherently a regional, rather than purely national, issue. Consequently, proactive cross-border management strategies – such as integrated early-warning systems, coordinated fuel break planning, and joint rapid response efforts – are critical for effectively mitigating fire risk throughout the Carpathian region. These considerations are further discussed in the Conclusion section.

### Model validation

Finally, to confirm the reliability of our model, we used an independent dataset containing active fire detections from NASA’s FIRMS database, covering the period from January 1, 2021, to May 1, 2025. After selecting high confidence detections (≥ 80% confidence), we retained 1,235 fire points. First, we compared the geographic coordinates of the negative control points to these fire detections at four-digit precision. There was no spatial overlap verifying that our negative controls were free from contamination by known fire events. Furthermore, the median great circle distance between each negative control point and the nearest recorded fire was approximately 12 km, and the mean distance was around 15 km, reinforcing their validity as genuine non-fire observations.

Next, we aimed to assess the predictive performance of our best-performing model. For each fire event recorded during the 2021–2025 validation period, we extracted the predicted fire probability within the corresponding H3 hexagon and compared it to the probabilities of hexagons without fire events during the same period. We found that the observed fire events occurred in hexagons with significantly higher predicted fire probabilities (Wilcoxon’s rank-sum test *p* < 2.2 × 10⁻¹⁶, see Fig. 6, panel d).

Finally, we investigated areas without fire events between 2010 and 2020 (*n* = 382,682) but were flagged by our model as highly vulnerable. All fire-free H3 hexagons from this period were divided into high-risk (*n* = 129,881) and low-risk (*n* = 252,801) groups using a predicted-probability threshold of 0.5. We then overlaid fire occurrences from 2021 to 2025 onto these hexagons. Fires were 5.14 times more frequent in the high-risk group than in the low-risk group (0.69% vs. 0.13%; Fisher’s exact test, *p* < 2.2 × 10⁻¹⁶). These results show that some regions appeared fire-resistant during 2010–2020 yet possessed climatic, anthropogenic, vegetational, and topographic characteristics that made them susceptible — a vulnerability our model successfully predicted based on these factors.

In sum, these results validate the integrity of the negative controls and demonstrate the robustness and predictive accuracy of the model, highlighting its effectiveness for future fire susceptibility assessments.

## Discussion

We compiled a standardised dataset and developed a comprehensive interpretable framework to assess fire susceptibility across the seven countries of the Carpathian region. The analysis relied on twenty-seven predictors from various sources, covering climatic, vegetation-related, topographic, and socio-economic factors. To refine our predictor set and improve overall interpretability, we applied both linear (VIF and Lasso) and non-linear (RFE) feature selection methods, as well as their combinations. Among the machine learning models optimised and trained, XGBoost performed best (test ROC AUC = 0.941) when using a combined feature selection approach – RFE applied to a VIF-filtered set – resulting in a final model with 19 predictors. We then applied SHAP interpretability framework at multiple scales to explore the influence of each predictor on model decisions. As expected, anthropogenic and vegetation-related variables were the most influential, while topographic predictors showed less impact but warrant further investigation. Finally, the resulting susceptibility maps identified areas with elevated fire risk, revealing eight major fire-prone clusters across the region. Below, we discuss our key findings, bridging both machine learning and environmental science perspectives.

### The interpretable machine learning pipeline

In environmental modelling, feature selection serves to reduce computational time, enhance model accuracy, and improve interpretability^84^. Given our initial set of twenty-seven predictors, evaluating all possible subsets was computationally infeasible, as it constitutes an NP-hard (nondeterministic polynomial) problem^85^. Therefore, we applied three complementary methods – VIF, Lasso, and RFE – and their combinations to isolate the most relevant variables. These methods have been similarly applied in related studies^25,33,86–92^. While most existing studies typically employ only one feature selection method^93,94^, our comprehensive approach explored a wider range of possible outcomes. Notably, none of the feature selection and machine learning methods identified the full set of predictors as the most effective for model performance, highlighting the value of targeted variable selection (see Table 1). The RFE method combined with a permissive VIF-filtered input set performed best, identifying a subset of 19 variables. Notably, except for the MLR, all tree-based algorithms selected subsets derived from RFE as optimal. Interestingly, both DRF and GBM models independently selected the same subset of 17 variables as their best choice (see Table 2). Our findings align with prior research indicating that wrapper methods, such as RFE, often yield smaller and more accurate predictor subsets^84^. Furthermore, the combination of a low-cost filter method (VIF) with a wrapper approach (RFE) proved highly effective, offering computational efficiency while effectively eliminating irrelevant variables in a two-stage process^84,95^. Additionally, our results suggest that recalculating variable importance at every iteration in RFE was not necessary – a practical consideration for future studies.

Fire predictability is high in the Carpathian region, with model performance comparable to that reported in similar studies^23,25,33,34^. ROC AUC values for the test sets varied across models (see Results and Supplementary Tables S4-S7). As expected, the weakest performance occurred when using a single-predictor subset – specifically, the RFE with the restrictive VIF set and rerank T setting. In contrast, the highest predictive accuracy was achieved using RFE-based subsets in the XGBoost-RFE-pVIF-F and DRF-RFE-rVIF-F models (see Table 2).

Tree-based models consistently outperformed MLR, regardless of the feature selection strategy used (see Supplementary Tables S4-S7). This result aligns with previous studies that compared both model types in similar contexts^30,31,33^. Beyond fire research, tree-based methods have also demonstrated strong predictive performance across diverse domains. For instance, in financial services, decision trees have been advocated for predicting commercial credit risk, complementing rather than replacing logistic regression^96^. Their intuitive structure, which closely mirrors human decision-making, makes them particularly appealing and defensible in high-stakes contexts such as loan approvals. In genetics, a comparative study^97^ applied four models - Logistic Regression, Logic Regression, Classification Trees, and Random Forest – and found Random Forest to be the most accurate. The authors emphasised not only its superior predictive power but also its capacity to handle high-dimensional datasets effectively. Perhaps the most compelling evidence comes from a large-scale benchmarking study^98^ that evaluated default implementations of Logistic Regression and Random Forest across 243 real-world binary classification datasets from the OpenML database. These models were tested without hyperparameter tuning, reflecting their widespread use as standard tools by practitioners with limited statistical expertise. The results showed that Random Forest outperformed Logistic Regression in 69% of the datasets based on accuracy, and in 72% based on AUC. Even in cases where Logistic Regression performed better, the performance gap was generally small.

While model performance remains important, there is a growing emphasis in machine learning on interpreting the outputs of complex black box models like XGBoost. This shift, driven by advances in interpretable and explainable AI (XAI), reflects an increasing demand to understand not just what a model predicts, but why. For researchers working with large datasets, especially in sensitive domains such as credit risk assessment, transparency and interpretability are essential for building trust in model decisions^99–101^. In our study, the SHAP framework enabled detailed interpretation of the factors driving fire predictions, both at the regional and local levels. It revealed notable spatial differences in variable behaviour – for example, the role of elevation in Borski county, Serbia, diverged from global trends. The SHAP summary plots (see e.g., Fig. 5, panel b) efficiently distill complex interactions into a clear visual format, offering a strong basis for further research. These insights pave the way for future research. For example, it would be valuable to investigate how variable importance shifts over time – such as, tracking whether NDVI gains predictive strength during peak fire season months.

### Human footprints and landscape features shaping fire susceptibility

All socio-economic (anthropogenic) variables in our initial feature set were included in the final model, and two of them – cropland density and distance from settlements – consistently ranked among the three most influential predictors (see Fig. 4, panel a). Their prominence confirms the decisive role of human activity in ignitions across the seven countries, echoing previous findings (see also Introduction). A 2021 synthesis for Czechia, Hungary, and Poland (2009–2018) attributed the vast majority of wildfires to intentional or accidental human causes^102^. The same report cited land use change in Romania, stubble burning in Serbia and other socio-economic drivers in Slovakia, although Ukraine was not assessed. In Polish forests, population density, building proximity and infrastructure are likewise key determinants of fire occurrence^35^.

Our results reinforce these patterns. Cultivated fields, settlement proximity and high population density emerged as the strongest anthropogenic predictors. Open field burning provides a clear mechanism: in Ukraine, agricultural fires dominated 2001–2003 and – despite their illegality – remain widespread, with flames easily spreading from croplands into forests^103–105^. Slovakian forests face similar pressures, with summertime stubble burning during droughts acting as a major ignition source^50^.

Vegetation attributes contributed equally to anthropogenic factors in our model. Vegetation-related variables ranked among the top predictors in all countries, except Serbia (see Fig. 5, panel b). Anthropogenic land cover change alters both NDVI and forest cover metrics: cultivated lands show low NDVI, whereas fallows register higher values^79^. Declining NDVI can also signal low fuel moisture, nutrient deficits, disease or repeated burning^46,80^. Conversely, high NDVI – and dense canopy cover – reflect healthy, more fire-resistant forests, a relationship captured by our model (see Fig. 5, panel b). Altitude further modulates this pattern. In Romania, rising temperatures lengthen the growing season and raise NDVI in high-altitude stands, but depress NDVI in lowland oak forests^106^. Our susceptibility map mirrors that contrast: the mountain core shows few high probability cells, whereas the foothills and lowlands form a broad belt of elevated susceptibility (see Fig. 6, panel b).

Topographical variables have a particularly strong influence on fire susceptibility in the southeastern Carpathians. Elevation, slope, and distance from water repeatedly rank among the leading predictors in Serbia, Romania, Hungary, and Ukraine (see Fig. 5, panel b). Topography shapes microclimate, fuel composition, and fire behaviour, as well as affecting solar radiation and water percolation rates^26^. Slope also affects evapotranspiration more strongly than temperature alone, altering local water balance^107^. Where surface water networks are sparse – particularly in Serbia, parts of Hungary and Romania – distance from streams becomes critical, reflecting the heightened flammability of dry landscapes (see Fig. 5, panel b). Indeed, proximity to water entered the top ten list in 44% of our counties (see Supplementary Figures S1), although it was not influential in Slovakia and Poland.

Water scarcity is an increasing concern in the identified region, as highlighted by previous studies^83,108^. The southeastern side of the Carpathians and Moldova have been found to experience the most pronounced climatic water deficits. Trend analysis revealed that these deficits are particularly acute along the West-East and North-South axes, with southeastern Romania facing the highest precipitation shortfalls^108^. This water deficit is further exacerbated by the lower climatological probability of drought recovery^109^, especially in the outer southeastern Carpathians. However, long-term hydro-climatic analysis of the region is well beyond the scope of this project but remains an excellent area for future research.

Due to the combined influence of these factors, fire susceptibility is concentrated in the lowlands and foothills, which cover roughly one-third of the study area and display probabilities ≥ 0.5 (see Fig. 6, panel b). Within this belt we delineated eight clusters using spatial clustering (see Fig. 6, panel b) that closely match the climatic vulnerability zones of^83^. As highlighted in the mentioned study, the dominance of certain fire-prone regions, particularly in the southern and eastern-facing Carpathians, may lead to misinterpretations of fire susceptibility in other areas. Focusing on smaller subregions can uncover regional characteristics that may not be visible or relevant at larger scales, e.g., in the case of Czechia where we did not identify any cluster.

### Limitation and future scope

Despite the study’s broad scope, some constraints should be noted. Although the model was trained with fire records and covariates spanning 2010–2020, the final susceptibility map was generated using covariate values sampled at the centroids of each H3 hexagon from March 2020, the peak fire month in the region (see Methods). Anchoring the predictions to this one-month snapshot means the map reflects conditions typical of early spring and may not capture susceptibility during other seasons or atypical fire years. Future work should move beyond this point-in-time snapshot by integrating time-resolved covariates and generating seasonally or even monthly updated, fully gridded maps.

The model was calibrated with eleven years of MODIS fire detections and collected covariates, ending in 2020. It therefore cannot include the unprecedented 2022–2023 fire seasons that swept much of Europe, and any novel patterns from those years remain unmapped. Future work should incorporate a larger time period for capturing model nuances more precisely.

In the current study, we did not make difference between forest fires and vegetation fires. While focusing only on either forest or vegetation fires could offer a more detailed understanding of fire development, narrowing our dataset to fires occurring exclusively in forested areas would have significantly reduced the size of our training data. Addressing this warrants future research.

The inclusion of certain predictors such as detailed vegetation composition, additional climatic parameters, and land use and land cover types (accounting for changes over time) could further enhance our dataset but at the time of writing our study, data were unavailable for the entire study area. In future work, the inclusion of temporal characteristics of fires can further contribute to model nuances. Additionally, the already collected dataset may also inherently suffer from additional uncertainties, e.g., occasional omissions or false alarms in fire detections or infrastructure vectors lacking year-specific updates, etc.

Addressing the above gaps suggests several avenues for refinement and expansion. First, coupling the framework with vegetation dynamics would provide valuable insights into how changes in phenological cycles over time affect fire susceptibility. These temporal signals could then be fed back into the predictive model to improve its accuracy.

Second, since climate change impacts extend well beyond fires, future work could explore the combination of fire susceptibility with other climate-sensitive hazards, such as floods, landslides, soil erosion, or drought. This multi-hazard integration could support genuinely integrated risk management.

Third, applying the enhanced workflow to other similar fire-prone region would test model generalisability and highlight region-specific adaptations. Together, these steps can turn the present static snapshot into a dynamic, multi-hazard decision tool tailored to the Carpathians – and adaptable to vulnerable landscapes elsewhere.

## Conclusion

This study delivers the first region-wide, interpretable assessment of vegetation fire susceptibility in the Carpathians. Leveraging an ensemble of tree-based learners (DRF, GBM, XGBoost) and SHAP post-hoc explanations, we distilled a twenty-seven-variable database into the nineteen most informative predictors and mapped ignition likelihood at a uniform 0.74 km^2^ resolution using the H3 hexagonal grid. The model performs on par with similar small-scale studies and reveals that roughly one-third of the mountain chain – 98,511 km^2^ – faces elevated (≥ 0.5) ignition probability, concentrated in eight clusters.

Across the seven Carpathian countries, human presence and vegetation condition dominate the ignition landscape. High cropland density and the proximity of fields and settlements consistently stood out as the strongest predictors, while vegetation-health proxies – the MODIS-derived NDVI indices and forest cover classes – were equally decisive. These findings reinforce earlier work attributing most wildfires in Central and Eastern Europe to direct or indirect human actions and underline land use management as a prime leverage point for mitigation. The role of climatic factors was evident too: warmer, drier periods, declining vegetation vigour, and unpredictable precipitation could, in fact, coincide with higher ignition probabilities, lending to partial support to the climate-fire paradigm. Because a formal attribution of long-term climate change effects would require dedicated temporal experiments beyond this study’s scope, we regard this link as suggestive rather than causal and flag it for future research.

Our dataset and the eight clusters identified make it possible to unravel the way socio-ecological mechanisms that elevate ignition probability. Because the SHAP-augmented model resolves feature contribution at county and even geographic coordinate-based scale, researchers can combine fine-grained field assessments with complementary interpretability techniques to trace the specific climatic, vegetation, topographic, and anthropogenic triggers operating in each cluster. Such micro-level diagnostics will enable foresters, fire service personnel, and municipal planners to integrate risk information into daily operations – whether through targeted fuel break placement, outreach campaigns, or joint training exercises – and to run “what if” scenarios that reveal presently low risk areas liable to become future hotspots under progressive warming.

To address the need for actionable insights, we have already proposed targeted policy recommendations^110^. Since all EU member states now enforce a strict stubble burning ban under Regulations 2021/2115 and 2116^111,112^, and Ukraine also prohibits open burning through its Code of Administrative Offences (Article 77 − 1) and Criminal Code (Articles 241 and 245)^113,114^, we recommend a synchronised seasonal moratorium in the high risk hexagons identified by our model. These bans should be supported by well-equipped rapid response inspection teams using satellite alerts and drones, along with subsidies for mechanical residue management to ensure effective and sustainable compliance^104^.

Second, our hexagon-level analysis shows that fire likelihood increases sharply where settlements, rails, and – to a lesser extent – road corridors intersect areas of low NDVI vegetation. Establishing and regularly maintaining fuel break buffers would help mitigate this compounded risk^115^. While no EU law mandates such buffers, the Common Agricultural Policy (CAP) Strategic Plans Regulation (2021/2115, Article 73) and its horizontal counterpart (2021/2116) offer dedicated funding and audit mechanisms to support them. In addition, Decision 1313/2013/EU on the Union Civil Protection Mechanism requires member states to address such measures in their national wildfire prevention plans^116,117^.

Third, our findings suggest southeastern Carpathian foothill areas where topographic factors – such as distance from water surfaces – correlate with increased fire occurrence. In these zones, restoring riparian buffers, afforesting hillsides with drought-resilient native species, and implementing small-scale water retention measures (e.g., ponds, reservoirs) would help maintain fuel moisture and reduce fire susceptibility^118^. These actions are now supported by EU law, notably through the Nature Restoration Regulation (in force since 18 August 2024), which sets binding targets for river connectivity, tree planting, and wetland recovery^119^. Collectively, these instruments provide Carpathian governments with both the legal mandate and financial tools to implement moisture-enhancing landscape measures in the high-risk areas identified by our study.

## Materials

### Study area

The Carpathian Mountains comprise a series of mountain chains forming a semicircle across Central and Eastern Europe, extending approximately 1,500 km from 44° to 50° North latitude and from 17° to 27° East longitude^120^. In delimiting our study area, we applied strict geographic criteria that follow the mountain chain itself and intentionally exclude the wider Carpathian macro-region^121^. Spanning an area of around 210,000 square kilometres, the range crosses seven countries: Czechia, Hungary, Poland, Romania, Serbia, Slovakia, and Ukraine^122,123^.

Characterised by a temperate continental climate with considerable local variability^124^, the Carpathians feature elevations predominantly between 750 and 2,500 m^120^. Vegetation patterns align closely with these altitudinal zones: mixed deciduous forests (pedunculate oak, lime, hornbeam) dominate lower elevations and intra-montane depressions (300–800 m), transitioning to European beech and silver fir in mid-mountain zones (600-1,450 m). Coniferous forests (Norway spruce, stone pine) prevail above 1,500 m, with sparse vegetation, such as mountain pine, dwarf juniper, and green alder, above the timberline^125^. Notably, the countries encompassing the Carpathians have recently experienced significant social, political, and economic transitions^126^.

### Administrative units

Administrative units within the study area were identified using the European Union’s (EU) Nomenclature of Territorial Units for Statistics (NUTS) classification. Due to spatial data availability, the NUTS 3 level was applied uniformly, except in Poland and Ukraine, where NUTS 2 was utilised. A comprehensive overview of administrative divisions is provided in Table 3.

**Table 3:** Administrative divisions of the study area as per the EU’s NUTS classification.

Country	Name of statistical unit	NUTS classification	Name of selected regions
Czechia	*kraj* (region, county)	NUTS 3	Moravskoslezský; Zlínsky
Hungary	*megye* (county)	NUTS 3	Borsod-Abaúj-Zemplén; Heves; Nógrád
Poland	*voivodeship* (province)	NUTS 2	Malopolskie (Lesser Poland); Slaskie (Silesian); Podkarpackie (Subcarpathian)
Romania	*judet* (county)	NUTS 3	Argeș; Bacău; Bistrița-Năsăud; Brașov; Buzău; Caraș-Severin; Covasna; Dâmbovița; Gorj; Harghita; Hunedoara; Maramureș; Mehedinți; Mureș; Neamț; Prahova; Sibiu; Suceava; Vrancea; Vâlcea
Serbia	*okrug/oblast* (district, county)	≈ NUTS 3	Borska; Branicevska
Slovakia	*kraj* (region, county)	NUTS 3	Banskobystrický; Kosický; Presovský; Trenčiansky; Žilinský
Ukraine	*oblast* (region, province)	≈ NUTS 2	Chernivtsi; Ivano-Frankivsk; Lviv; Zakarpattia

### Dependent variable

The dependent variable – fire pixels – was sourced from NASA’s FIRMS MODIS aboard Terra and Aqua satellites (https://firms.modaps.eosdis.nasa.gov/). MODIS detects thermal anomalies, representing fires with approximately 1 km spatial resolution. The instrument documentation clarifies that the resulting fire pixels may not necessarily align with the exact fire size or number^127,128^. We represented these data as point coordinates centred within each pixel to reduce computational load and eliminate the need to standardise variable resolutions^86,129–132^.

Data from 2010 to 2020 were organised by year and country. Following common research practice^133^, only fire occurrences with a confidence value of at least 80% were included. Using spatial vector data from the Global Administrative Areas Database (GADM, version 2.5 with a release date of July 2015) (https://gadm.org/index.html), we identified 5,173 fire points. Most (5,170) were concentrated within single administrative regions, with three points on regional borders attributed to Ukraine’s Lviv region due to its higher incidence of fires. Each identified fire location was coded as 1.

### Generation of control points

Using a stratified random sampling method, we generated an equal number of non-fire control points, coded as 0, ensuring their spatial and temporal characteristics matched those of the fire coordinates. To maintain local spatial independence and prevent adjacency to fire pixels, we implemented the Vincenty spherical method^134^, defining a minimum distance of 5,000 m between fire and non-fire points^135^.

Due to a small sample size in the regions of Czechia and Slovakia, we merged the regions of the respective countries and treated them as a single observation area. This consolidation resulted in identifying 129 non-fire points in Czechia and 277 in Slovakia. We generated the non-fire coordinates with 5-digit accuracy, similar to fire coordinates, and handled missing data in the same way as for fire points (see Explanatory variables subsection).

After data collection and generation, a data frame containing all fire and non-fire points (*n* = 10,346) was created, incorporating fire/non-fire labels, predictor columns (twenty-seven covariates), and additional metadata (e.g., country name, region name). The balanced dataset ensures that the machine learning models accurately categorise points into their respective classes^136^.

### Explanatory variables

A comprehensive dataset of 26 explanatory variables was compiled, categorised into 11 climatic, 3 vegetation, 5 topographic, and 7 socio-economic factors, chosen based on literature relevance and data availability. Table 4 presents a summary of each variable.

**Table 4:**
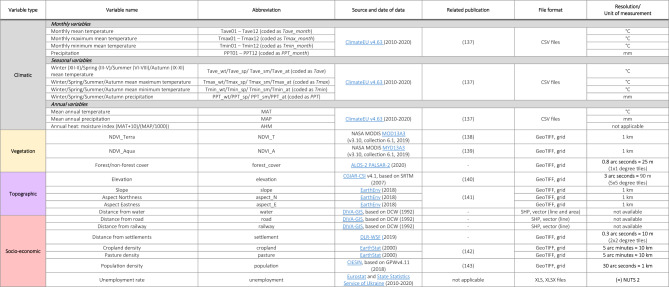
Explanatory variables selected for analysis. Variables are grouped and colour-coded as in Tables 1; Fig. 5.

### 1. Climate variables

The selection of meteorological variables posed challenges due to limited data availability and data inconsistency. At the time of writing our study, websites hosting meteorological databases such as WorldClim (https://worldclim.org) and CarpatClim (http://www.carpatclim-eu.org/pages/home/) lacked adequate data for our predefined timeframe. Additionally, data from various meteorological stations supplied by the European Climate Assessment & Dataset (https://www.ecad.eu) lacked standardised data across countries. Consequently, interpolated climatic data from the ClimateEU software package (https://sites.ualberta.ca/~ahamann/data/climateeu.html) were utilised^137^. We selected 4 monthly, 4 seasonal, and 3 annual climatic variables, covering temperature and precipitation patterns.

During this process, we stored fire coordinates with elevation values in CSV files and loaded them into the ClimateEU software. The software generated climatic data in separate downloadable CSV files, which were then matched temporally to the corresponding fire and control points. Specifically, annual climatic variables were aligned with the corresponding year of each fire and control point; seasonal climatic data were matched to the exact year and season; and monthly data were aligned with the precise year and month associated with each point. As advised^144^, elevation data accompanied fire and control coordinates to enhance climatic accuracy. Descriptive statistics for the climatic data are provided in Supplementary Table S8.

### 2. Vegetation variables

Vegetation characteristics were represented by forest/non-forest cover data from the Advanced Land Observing Satellite (ALOS) Research and Application Project (https://www.eorc.jaxa.jp/ALOS/en/dataset/fnf_e.htm), utilising the 25 m resolution PALSAR-2 product, version 2, and NDVI values, from NASA MODIS Terra and Aqua satellites (https://lpdaac.usgs.gov/products/mod13a3v061/, https://lpdaac.usgs.gov/products/myd13a3v061/).

#### Forest cover

Forest cover was classified according to FAO definition of “forest”, denoting tree-covered land with an area larger than 0.5 ha and a canopy cover exceeding 10%. This results in a categorical variable with four values: 1 meaning a forested area with a canopy cover of 90% or more; 2 meaning a forested area with a canopy cover between 10% and 90%; 3 meaning non-forested area; and 4 meaning watered surface. The forest cover value for each point was extracted using the corresponding spatial raster file. Our dataset revealed 316 fire points in Category 1; 558 in Category 2; and 51 in Category 4; with the majority of coordinates (4,248) falling into Category 3, indicating a predominance (> 82%) of fire occurrences in sparsely forested areas.

#### NDVI values

In terms of NDVI values, the Terra satellite – following a morning orbit from North to South – crosses the Equator at approximately 10:30 am, whereas Aqua, with its afternoon orbit from South to North, reaches the Equator at around 1:30 pm. For data extraction, we used the Application for Extracting and Exploring Analysis Ready Samples (AppEEARS, v3.5) to obtain the MOD13A3 and MYD13A3 Collection 6.1 products, which provide NDVI data at a spatial resolution of 1 km and a monthly temporal resolution. NDVI values range from − 1 to + 1: values close to −1 typically indicate water surfaces, values near + 1 suggest dense vegetation, and values around 0 generally represent urbanised areas. In our dataset, NDVI values range from − 0.01 to 0.88 (Terra) and from 0.06 to 0.89 (Aqua), with median values of 0.46 and mean values of 0.47 for both satellites. As with the climatic data, NDVI values were matched temporally to the fire and control points based on the closest date.

### 3. Topographic variables

Five topographic variables, including elevation, slope, aspect (eastness and northness), and proximity to water bodies, were sourced to understand their influence on fire occurrences.

#### Elevation

For elevation, we used the reprocessed version of NASA’s Shuttle Radar Topographic Mission (SRTM) digital elevation model, as provided by CGIAR-CSI version 4.1 (based on SRTM 2007) (https://srtm.csi.cgiar.org). This version, processed by CGIAR-CSI GeoPortal, offers global coverage without data voids and a spatial resolution of 90 m. Elevation values were extracted from the raster layer for each point. Analysis showed a minimum elevation of 14 m and a maximum of 2,256 m, with a median elevation of 255 m, indicating most fire points were located at lower elevations. The mean elevation was 299.6 m, suggesting a right-skewed distribution of fire points.

#### Slope

For slope and aspect variables, we relied on a standardised and global multivariate product provided by^141^ which incorporates data from the 250 m Global Multi-resolution Terrain Elevation Data 2010 (GMTED) and the near-global 90 m SRTM 4.1dev (https://www.earthenv.org/topography). The authors generated a suite of elevation-derived topographic variables across various spatial resolutions. For our purposes, we used slope and aspect data at a 1 km resolution with median aggregation from GMTED sources. Values for each fire and control point were extracted from these raster datasets. The slope data exhibited a highly right-skewed distribution, with most fire events occurring on gentle slopes of less than 5%.

#### Aspect variables

In addition to slope, which reflects the magnitude of elevation change, the aspect provides further insight into the terrain’s orientation – an important factor in fire susceptibility. Therefore, we used the derived northness and eastness variables, which are transformations of slope and aspect (see^141^, for calculation details). In the Northern Hemisphere, the value of northness ranges from − 1 (South-facing) to + 1 (North-facing), with 0 indicating an East-West orientation. Similarly, for eastness, the value range is between − 1 (West-facing) and + 1 (East-facing), where 0 represents neither a West nor East-facing direction, but a South-North exposition. Our dataset shows a slight predominance of fires on South- and East-facing slopes, with median values of −0.03 and 0.02, respectively.

#### Distance from water bodies

To assess the proximity of fires to water bodies, we obtained shapefiles of rivers and lakes in the Carpathian region from the DIVA-GIS database (https://www.diva-gis.org), along with road and railway infrastructure (see Socio-economic variables subsection). Using great-circle distance calculations, we determined the distance between fire points and the nearest water body. The median was slightly over 2 km, with a mean distance exceeding 2.6 km.

### 4. Socio-economic variables

#### Distance from infrastructural networks

We acquired road and railway infrastructure data from the same DIVA-GIS source as the hydrological data. Great-circle distances were calculated for both types of infrastructure. The median distance to the nearest road was slightly over 2 km, with an average of approximately 2.5 km. For the railway network, the median distance was 4.3 km and the mean was 6.2 km.

#### Distance from settlements

Settlement data were sourced from the World Settlement Footprint 2019 dataset, developed by the German Aerospace Center (DLR-EOC Geoservice), with a spatial resolution of 10 m (https://geoservice.dlr.de/web/maps/eoc:wsf). Using this dataset, we calculated Vincenty spherical distances between each point and the nearest settlement. The results indicated a right-skewed distribution, with a median distance of 750 m and a mean of approximately 935 m, suggesting that most fire occurrences were located close to human settlements.

#### Cropland and pasture densities

We used cropland and pasture density data from the EarthStat database (http://www.earthstat.org/cropland-pasture-area-2000/), which combines inventory data with satellite-derived land cover estimates. The values range from 0 to 100%. While extracting data for fire and non-fire points from the relevant raster tiles, we encountered 32 instances of missing values. These values were substituted with the ones from the nearest raster tiles with non-missing values.

#### Population density

Population density data, measured in persons per square kilometre, were obtained from the Gridded Population of the World (GPW) version 4.11, hosted by NASA’s Socioeconomic Data and Application Center (SEDAC) (https://sedac.ciesin.columbia.edu/data/set/gpw-v4-population-density-rev11). The data were provided in GeoTIFF format at a 1 km resolution. We analysed the datasets for the years 2010, 2015, and 2020 and selected the value closest in time to each fire and control point. The median population density was 66 persons/km^2^, while the mean reached 147 persons/km^2^, indicating right-skewed distribution.

#### Unemployment rate

Unemployment rates at the NUTS 2 level were compiled using official reports from the Ukrainian National Statistical Office and Eurostat. These publicly available datasets can be accessed at http://www.ukrstat.gov.ua/operativ/operativ2009/rp/rp_reg/reg_e/arh_rbn_e.htm and https://ec.europa.eu/eurostat/databrowser/view/tgs00010/default/table?lang=en. The statistics pertain to the working population aged 15 and above. Due to data constraints, Ukrainian unemployment rates were taken from 2010 to 2018 and refer to individuals aged 15 to 70. We identified 88 missing values for Serbia between 2010 and 2012. These were supplemented with national-level data obtained from the Serbian Statistical Office (https://data.stat.gov.rs/Home/Result/24000100?languageCode=en-US). As with other variables, unemployment rates were matched to the corresponding region and year of the fire and control points. All referenced websites were accessed in August 2022.

## Methods

### Data Preparation and handling

In a linear world, multicollinearity – when two or more variables are highly correlated – is a condition to be avoided, as it makes it difficult to isolate the individual effects of each variable on the dependent outcome^145^. Our correlation analysis of the original 26 predictors identified significant multicollinearity, particularly among monthly and seasonal climatic variables (see Supplementary Figures S3). To address this, we combined highly correlated predictor pairs (*Tmax_month* with *Tmin_month* and *Tmax* with *Tmin*) into two new variables: *Trange_month* and *Trange_seasonal*, representing monthly and seasonal temperature ranges, respectively. Additionally, we transformed the categorical predictor (*forest_cover*) into dummy variables. Following these adjustments, the final dataset comprised 27 refined predictors suitable for subsequent modelling stages.

#### Data splitting

Our dataset consists of 10,346 observations, evenly split between 5,173 fire points and 5,173 control (non-fire) points, collected for the period from 2010 to 2020. Initially, we split the data into two main subsets: an 80% training set and a 20% testing set, with the latter reserved exclusively for evaluating the performance of our machine learning models. To ensure unbiased model training and evaluation, the training set was further divided into an 80:20 ratio. The larger subset was utilised during feature selection, which was evaluated on the smaller subset. We applied 10-fold cross-validation during both feature selection and model training stages, an established technique in the field^25,33,86^, to mitigate overfitting and enhance model reliability. All datasets were scaled separately.

### Feature selection approach

**Fig. 7:**
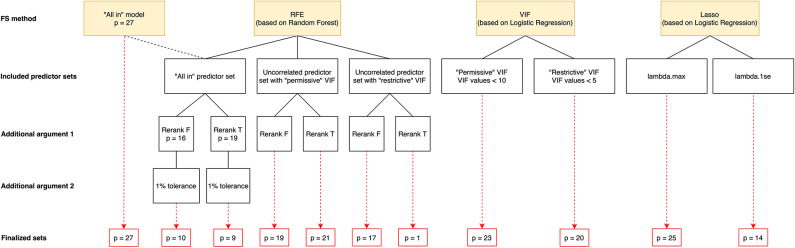
Summary of feature selection methods and their resulting feature subsets. Specific selection criteria and arguments are also indicated for each method (see text for details). *p* indicates the number of variables in each subset.

Given the complexity and multicollinearity inherent in our initial set of 27 predictors, effective feature selection was crucial to enhance model interpretability and performance. We adopted three distinct feature selection methods representative of the major categories: Variance Inflation Factor (VIF) from filter methods, Recursive Feature Elimination (RFE) from wrapper methods, and Least Absolute Shrinkage and Selection Operator (Lasso) from embedded methods. Each method and their combinations provided unique insights into predictor importance and improved the robustness of our feature selection process. We summarized the methods and their resulting feature subsets in Fig. 7.

#### Variance inflation factor (VIF)

VIF assesses collinearity among predictors in linear regression models by measuring how much the variance of an estimated regression coefficient increases due to collinearity^146^. It is calculated by comparing the variance of the coefficient when all variables are included in the model to the variance when only the examined variable is included^37^. A VIF of 1 indicates that the variable is not correlated with others in the model, and theoretically, its upper range can be infinite. However, there is not a universally agreed-upon threshold for what constitutes a high or low VIF, the rule of thumb is generally around 5 to 10 as acceptable^147^. These reference values were used to determine the subsets in our analysis using the *car* R package.

Supplementary Table S9 shows the summary of the VIF calculation process. Starting with all variables, we iteratively removed predictors with the highest VIF (marked with red), recalculating at each step, until achieving subsets with acceptable collinearity thresholds below 10 (permissive set) and 5 (restrictive set) (see Supplementary Figures S4).

#### Recursive feature elimination (RFE)

RFE iteratively removes the least important predictors based on model performance, gradually refining the subset to an optimal predictor group^148^. We implemented RFE with Random Forest as the underlying algorithm using the *caret* R package. We ran several variations of the algorithm by including only correlated and also uncorrelated predictors (as determined by the VIF analysis), recalculating variable importance after each iteration (rerank: T and F on Fig. 7), and applying a tolerance level of 1% on the correlated set to achieve balanced model complexity and predictive accuracy.

#### Least absolute shrinkage and selection operator (Lasso)

Lasso performs variable selection and regularisation by applying an L1 penalty to regression coefficients, shrinking some coefficients to zero in case of a sufficiently large $\:{\uplambda\:}$ value, thus, effectively eliminating less relevant predictors^73,149^. This regularisation technique is particularly useful to avoid overfitting as the algorithm encourages a sparse model with fewer predictors^150^. If $\:{\uplambda\:}$ is equal to zero, it means all variables are considered without any shrinkage penalty added to the model, so that results are equal to the ones obtained from a simple linear regression model. If $\:{\uplambda\:}$ is set to infinite, no variables are considered at all, except the intercept. Thus, choosing the optimal value of $\:{\uplambda\:}$ is critical^37^. We determined two lambda values through 10-fold cross-validation: $\:{\lambda\:}_{max}$, which resulted in the maximum cross-validated Receiver Operating Characteristics – Area Under the Curve (ROC AUC) value, and $\:{\lambda\:}_{1se}$ which resulted in an AUC which is one standard error from the maximum AUC value. For this, we used the *glmnet* R package. Supplementary Figure S5 shows the obtained $\:\lambda\:$ values and the associated number of predictors.

### Machine learning model development

We trained and optimised four supervised machine learning algorithms – Distributed Random Forest (DRF), Gradient Boosting Machine (GBM), eXtreme Gradient Boosting (XGBoost), and Multiple Logistic Regression (MLR) – within the H2O-3 environment (H2O.ai, 2023, R package version 3.44.0.3, https://github.com/h2oai/h2o-3). Each algorithm underwent extensive parameter tuning via a random grid search across hyperparameter combinations on 80% of the entire dataset. ROC AUC was used as the primary stopping metric to halt training when improvements became marginal (< 0.1%) for five consecutive scoring rounds. The maximum runtime per model subset was capped at two hours to ensure computational efficiency.

#### Model evaluation

Model performance was assessed using several metrics suitable for balanced binary classification problems, including accuracy, sensitivity, specificity, precision, F1 score, and ROC AUC. These comprehensive evaluation criteria ensured a holistic understanding of each model’s predictive capabilities.

#### Distributed random forest (DRF)

DRF is the default Random Forest implementation in H2O, enhancing Breiman’s original Random Forest algorithm with a distributed computing strategy to significantly increase execution speed^38^. The term “distributed” highlights the approach of building and reading trees in parallel, distributed across clusters or nodes, with trees stored in memory in a compressed manner. Each node retains its portion of the training data, and the algorithm exchanges necessary information between these predefined nodes^151^. By constructing multiple trees using random samples of predictors and resampling the training data observations – a technique known as bagging – DRF effectively overcomes the high variance and poor prediction accuracy associated with a single decision tree. For optimising DRF, we used the parameters listed in Supplementary Table S5.

#### Gradient boosting machine (GBM)

GBM is a forward-learning ensemble method that combines the strengths of gradient-based optimisation and boosting^152^. This classifier works by sequentially combining shallow decision trees, known as base or weak learners. In GBM, each tree depends on the previous ones, and new trees are trained on modified versions of the original dataset. This dependency allows each subsequent tree to correct the errors of its predecessors. The term “gradient” in GBM refers to the use of the gradient descent optimisation algorithm, which iteratively adjusts parameters to minimise the cost function^153^. Specifically, GBM uses gradient descent to minimise the loss function. The learning rate, also known as shrinkage, controls the contribution of each tree to the final model.

H2O uses a distributed approach to build GBM trees. Each data row is assigned to a tree node, with a temporary vector storing node IDs. H2O scans rows and creates histograms for each node to decide how to split the data. Initially, all rows start at node 0, and decisions are made based on statistical tests of homogeneity. As the tree grows layer by layer, rows are reassigned to new nodes based on conditions set at each level. This method allows rapid parallel processing of large datasets by leveraging multiple CPUs, therefore accelerating model building, although it can potentially increase communication demands for deep trees^152^. For optimising GBM, we used the parameters listed in Supplementary Table S6.

#### eXtreme gradient boosting (XGBoost)

XGBoost builds on the foundation of gradient boosting while enhancing computational speed and model performance^154^. It introduces several key improvements, such as loss function normalisation, regularisation techniques, and support for both decision trees and linear classifiers. One of its core innovations is the use of Taylor series expansion, which incorporates both the first derivative (gradient) and second derivative (Hessian) of the loss function for more accurate optimisation. To improve performance and prevent overfitting, XGBoost includes additional regularisation terms in its objective function and applies techniques like dropout^153^. It also uses strategies such as shrinkage (reducing the learning rate) and feature subsampling, where only a subset of features is used to build each tree, further helping to control overfitting. The algorithm’s implementation in H2O is similar to GBM, and for optimisation, the parameters outlined in Supplementary Table S7 were used.

#### Multiple logistic regression (MLR)

In addition to tree-based models, we also introduced a simpler regression-based method as a benchmark. Logistic Regression, part of the generalized linear models (GLM) suite, can be used in H2O for predicting a binary response variable with multiple predictors. Instead of modeling the response variable directly, Logistic Regression models the probability that the response variable $\:Y$ belongs to one of two categories, such as TRUE (fire occurrence, coded as 1) or FALSE (fire non-occurrence, coded as 0). Mathematically, it is an extension of linear regression based on the logit or log-odds function, the natural logarithm of an odds ratio. Its inverse is the sigmoid or logistic function, which takes any real number and projects it onto the $\:\left[\text{0,1}\right]$ range, representing the probability of an event occurring. The model was trained using the maximum likelihood estimation to find the optimal coefficients that minimised the difference between the predicted probabilities and the actual binary outcomes.

### Model interpretability

To interpret our best-performing model, we utilised the SHapley Additive exPlanations (SHAP) framework within H2O. SHAP, grounded in Shapley values from cooperative game theory, quantifies the contribution of each feature to the model’s predictions, offering clear and interpretable insights into both feature importance and the direction of their influence. The Shapley value itself represents the average marginal contribution of a feature value across all possible combinations (coalitions) of features^99,155^. As the exact computation of Shapley values is highly demanding, SHAP uses efficient approximation techniques to make the process more practical.

Widely recognised as a model-agnostic post-hoc explanation method, SHAP is rooted in solid mathematical theory. However, it is important to note that it does not imply causation; rather, it identifies which variables influence the model’s decisions and in which direction. In our study, we applied SHAP both globally considering all points across the Carpathian region or in a given county, and locally – at the level of one coordinate – allowing us to uncover fine-scale spatial patterns and the varying impact of predictors.

### Fire susceptibility mapping

One of the primary objectives of our research was to project fire ignition patterns across the Carpathian region. To achieve this, fire susceptibility was visualised using the H3 hierarchical hexagonal geospatial indexing system, with the *h3jsr* R package (https://cran.r-project.org/web/packages/h3jsr/index.html). Originally developed by Uber in 2018, H3 divides the Earth’s surface into hexagonal grids, offering several advantages well beyond its transportation origins (https://h3geo.org)^156–158^. The hexagonal grid structure ensures that neighbouring cells are equidistant, making adjacent neighbour queries more efficient. Additionally, more relevant for the current study, H3’s icosahedron-based projection minimises spatial distortions when mapped onto the Earth.

Leveraging our best-performing predictive model, we estimated fire probabilities for hexagonal grid cells (each approximately 0.74 km^2^). To calculate these probabilities, we used the most recent data available on the central point coordinate of each H3 cell. Specifically, climatic conditions from March 2020, aligning peak fire season, informed this mapping process. The resultant maps highlighted regions of elevated fire probability using spatial clustering, enabling the identification of potential fire risk clusters across all seven Carpathian countries. For this, we used the *spdep* R package.

Finally, we used predicted probability values for calculating a post-hoc global Moran’s $\:I$ on the Pearson-standardised residuals (H3 grid, resolution 8, queen contiguity). The value was 0.18 (*p* < 2.2e-16), indicating weak positive spatial autocorrelation. This suggests that using a 5-km buffer during the generation of negative controls did not fully remove spatial autocorrelation. However, the strong validation results (see Results - Model validation) demonstrate that this low level of spatial autocorrelation has minimal impact on model performance. Future research could further mitigate spatial autocorrelation by adding explicit spatial covariates.

## Electronic supplementary material

Below is the link to the electronic supplementary material.

Supplementary Material 1

## Data Availability

All MODIS data and other explanatory variables used in this study are publicly available online without access restrictions or the need for special permissions. The original data, the compiled dataset, and all associated R code are available in the Harvard Dataverse repository (10.7910/DVN/MGSGTF). The dataset generated and/or analysed during the this study, along with the R code, is also available in the GitHub repository (https://github.com/mame-91/fire_susceptibility_r). For additional information, please contact the corresponding author.
