# Supplementary material for: Fire susceptibility assessment in the Carpathians using an interpretable framework
Source: Sci Rep. 2025 Aug 18;15:30207. doi: 10.1038/s41598-025-10296-4 (PMC12361508; doi:10.1038/s41598-025-10296-4)
Supplement: Supplementary file 1 — Supplementary Material 1 [file 41598_2025_10296_MOESM1_ESM.pdf]

Supplementary materials for research article

Melinda Manczinger[1, *], László Kovács[2], Tibor Kovács[3]

Fire Susceptibility Assessment in the Carpathians Using an Interpretable Framework

Supplementary Table S1

Year	Fire points	Non-fire points
2010	n = 268	n = 268
2011	n = 623	n = 623
2012	n = 991	n = 991
2013	n = 419	n = 419
2014	n = 398	n = 398
2015	n = 581	n = 581
2016	n = 428	n = 428
2017	n = 444	n = 444
2018	n = 233	n = 233
2019	n = 392	n = 392
2020	n = 396	n = 396

Supplementary Table S1. Basic descriptive statistics for fire and non-fire points in the database.

Supplementary Table S2

Metric	Calculation	RFE-all-in-F n = 10	RFE-all-in-T n = 9	RFE-pVIF-F n = 19	RFE-pVIF-T n = 21	RFE-rVIF-F n = 17	RFE-rVIF-T n = 1	LASSO-lambda.min n = 25	LASSO-lambda.1se n = 14	Permissive VIF n = 23	Restrictive VIF n = 20
TP		723	718	745	741	745	475	691	692	684	683
TN		667	666	679	675	688	358	626	614	601	595
FP		161	162	149	153	140	470	202	214	227	233
FN		105	110	83	87	83	353	137	136	144	145
SUM		1656	1656	1656	1656	1656	1656	1656	1656	1656	1656
Accuracy	(TP+TN)/ (TP+TN+FP+FN)	0.839371981	0.835748792	0.859903382	0.855072464	0.865338164	0.503019324	0.795289855	0.788647343	0.775966184	0.77173913
Kappa		0.6787	0.6715	0.7198	0.7101	0.7307	0.006	0.5906	0.5773	0.5519	0.5435
Sensitivity/ Power/ Recall/TPR	TP/(TP+FN)	0.873188406	0.867149758	0.899758454	0.894927536	0.899758454	0.573671498	0.834541063	0.835748792	0.826086957	0.824879227
Specificity/TNR	TN/(TN+FP)	0.805555556	0.804347826	0.820048309	0.815217391	0.830917874	0.43236715	0.756038647	0.741545894	0.725845411	0.718599034
PPV/Precision	TP/(TP+FP)	0.817873303	0.815909091	0.833333333	0.82885906	0.84180791	0.502645503	0.773796193	0.763796909	0.750823271	0.745633188
NPV	TN/(TN+FN)	0.863989637	0.858247423	0.891076115	0.885826772	0.892347601	0.503516174	0.820445609	0.818666667	0.806711409	0.804054054
F1	(2*Precision* Recall)/ (Precision + Recall)	0.844626168	0.840749415	0.865272938	0.860627178	0.869819031	0.535815003	0.803021499	0.798154556	0.786658999	0.783256881
ROC AUC (test)		0.9231	0.9159	0.9314	0.933	0.9348	0.4398	0.859	0.8551	0.8392	0.8396

Supplementary Table S2. Evaluation metrics for the 10 different subsets used.

Supplementary Table S3

FS algorithm	Specified setup	Training runtime
VIF	"Permissive" VIF: iterative elimination of predictors until variables with VIF value < 10 is reached (n = 23)	<1 min
VIF	"Restrictive" VIF: iterative elimination of predictors until variables with VIF value < 5 is reached (n = 20)	<1 min
RFE	"All in" predictor set with rerank FALSE (n = 10) after 1% tolerance is applied	~46 min
RFE	"All in" predictor set with rerank TRUE (n = 9) after 1% tolerance is applied	~44 min
RFE	Uncorrelated predictor set (= "permissive" VIF) with rerank FALSE (n = 19)	~34 min
RFE	Uncorrelated predictor set (= "permissive" VIF) with rerank TRUE (n = 21)	~35 min
RFE	Uncorrelated predictor set (= "restrictive" VIF) with rerank FALSE (n = 17)	~27 min
RFE	Uncorrelated predictor set (= "restrictive" VIF) with rerank TRUE (n = 1)	~26 min
Lasso	Lasso predictor set identified with λ_min (n = 25)	<1 min
Lasso	Lasso predictor set identified with λ_1se (n = 14)	<1 min

Supplementary Table 3. Runtimes of feature selection methods used in the analysis.

Supplementary Tables S4-S7

[Supplementary Table S4]

Multiple Logistic Regression results

Algorithm name	Argument name	Argument description	Parameter space	"All in" n = 27	"Permissive" VIF n = 23	"Restrictive" VIF n = 20	RFE "all in" set with rerank F n = 10	RFE "all in" set with rerank T n = 9	RFE "permissive" VIF set with rerank F n = 19	RFE "permissive" VIF set with rerank T n = 21	RFE "restrictive" VIF set with rerank F n = 17	RFE "restrictive" VIF set with rerank T n = 1	Lasso lambda.min n = 25	Lasso lambda.1se n = 14
MLR	*Without hyperparameter optimization: family = "binomial", link = "logit", regularization = None*													
	Summary		Number of models built	1	1	1	1	1	1	1	1	1	1	1
			Model ID	MLR_full	MLR_pVIF	MLR_rVIF	MLR_RFE_all_f	MLR_RFE_all_t	MLR_RFE_pvif_f	MLR_RFE_pvif_t	MLR_RFE_rvif_f	MLR_RFE_rvif_t	MLR_LASSO_min	MLR_LASSO_1se
			ROC AUC (training after 10-fold CV)	0.860	0.843	0.841	0.852	0.851	0.837	0.843	0.841	0.773	0.860	0.860
			ROC AUC (test)	0.857	0.847	0.844	0.850	0.850	0.848	0.847	0.844	0.791	0.856	0.857

Supplementary Table S4. The MLR model results for each subset. The highest AUC values for both the training and test sets are highlighted in green, while the best subset is circled in red. In cases where results are the same, the smallest possible subset is chosen as the best subset.

[Supplementary Table S5]

Distributed Random Forest results

Algorithm name	Argument name	Argument description	Parameter space	"All in" n = 27	"Permissive" VIF n = 23	"Restrictive" VIF n = 20	RFE "all in" set with rerank F n = 10	RFE "all in" set with rerank T n = 9	RFE "permissive" VIF set with rerank F n = 19	RFE "permissive" VIF set with rerank T n = 21	RFE "restrictive" VIF set with rerank F n = 17	RFE "restrictive" VIF set with rerank T n = 1*	Lasso lambda.min n = 25	Lasso lambda.1se n = 14
DRF	*mtries*	The number of features randomly sampled as candidates at each split.	round $(\sqrt{p}) \pm 2$	5	5	4	2	3	4	5	4	1	5	4
	ntrees	The number of trees built.	5, 50, 100, 200, 500	500	500	500	500	200	500	500	500	500	500	500
	max_depth	The number of splits each tree is allowed to make.	3, 5, 10, 20, 30	30	30	30	30	30	30	30	30	30	30	30
	col_sample_ rate_per_tree	Relative change of the column sampling rate for each tree.	0.5, 0.9, 1	0.5	0.5	0.5	0.5	0.5	0.5	0.5	0.5	0.5	0.5	0.5
	sample_rate	Training data (rows subsampled) used to train each tree.	0.5, 0.632, 0.8, 0.95, 1	0.95	0.95	0.95	1	1	0.95	0.95	0.95	0.5	0.95	0.95
	Summary		Number of models built	74	76	80	193	192	183	81	176	237	79	53
			Best model ID	full_model_7	pVIF_model_7	rVIF_model_7	RFE_all_f_ model_119	RFE_all_t_ model_110	RFE_pvif_f_ model_7	RFE_pvif_t_ model_7	RFE_rvif_f_ model_7	RFE_rvif_t_ model_113	LASSO_min_ model_7	LASSO_1se_ model_7
			ROC AUC (training after 10-fold CV)	0.943	0.946	0.948	0.942	0.940	0.948	0.947	0.951	0.922	0.943	0.936
			ROC AUC (test)	0.933	0.939	0.939	0.922	0.911	0.939	0.939	0.940	0.652	0.935	0.923

Supplementary Table S5. Hyperparameter optimization in DRF. For each subset, the parameter values for the best models are determined by maximizing the ROC AUC. The highest AUC values for both the training and test sets are highlighted in green, while the best subset is circled in red. *Note: In the subset with n = 1, there were 483 failed models due to invalid mtries options, as only one variable is present in the subset. In all other cases, the modeling procedure was successful without any failed models.

[Supplementary Table S6]

Gradient Boosting Machines results

Algorithm name	Argument name	Argument description	Parameter space	"All in"<br>n = 27	"Permissive" VIF<br>n = 23	"Restrictive" VIF<br>n = 20	RFE "all in" set with rerank F<br>n = 10	RFE "all in" set with rerank T<br>n = 9	RFE "permissive" VIF set with rerank F<br>n = 19	RFE "permissive" VIF set with rerank T<br>n = 21	RFE "restrictive" VIF set with rerank F<br>n = 17	RFE "restrictive" VIF set with rerank T<br>n = 1	Lasso lambda.min<br>n = 25	Lasso lambda.1se<br>n = 14
GBM	*col_sample_rate*	The number of features randomly sampled as candidates at each split.	seq(0.1,0.5,0.1), seq(0.55, 1, 0.05)	0.75	0.8	0.8	0.75	0.75	0.8	0.8	0.75	0.8	0.8	0.75
	ntrees	The number of trees built.	5, 50, 100, 200, 500, 1000, 5000, 10000	1000	500	500	1000	1000	500	500	1000	500	500	1000
	max_depth	The number of splits each tree is allowed to make.	3, 5, 10, 20, 30	30	30	30	30	30	30	30	30	30	30	30
	col_sample_rate_per_tree	Relative change of the column sampling rate for each tree.	0.5, 0.9, 1	0.5	0.5	0.5	0.5	0.5	0.5	0.5	0.5	0.5	0.5	0.5
	sample_rate	Training data (rows subsampled) used to train each tree.	0.5, 0.632, 0.8, 0.95, 1	1	0.95	0.95	1	1	0.95	0.95	1	0.95	0.95	1
	col_sample_rate_change_per_level	Relative change of the column sampling rate for every level in each tree.	0.9, 1, 1.1	1	0.9	0.9	1	1	0.9	0.9	1	0.9	0.9	1
	min_rows	Minimum number of observations for a leaf to split.	5, 10, 20	10	5	5	10	10	5	5	10	5	5	10
	min_split_improvement	Minimum relative improvement in order for a split to happen.	0, 1e-8, 1e-6, 1e-4	0	0	0	0	0	0	0	0	0	0	0
	histogram_type	Type of histograms to use to find the optimal split points.	AUTO, UniformAdaptive, Random, QuantilesGlobal, RoundRobin	Uniform Adaptive	Quantiles Global	Quantiles Global	Uniform Adaptive	Uniform Adaptive	Quantiles Global	Quantiles Global	Uniform Adaptive	Quantiles Global	Quantiles Global	Uniform Adaptive
	nbins	Number of bins for split-finding for numeric columns.	2, 16, 20, 1024	16	1024	1024	16	16	1024	1024	16	1024	1024	16
	nbins_cats	Number of bins for split-finding for categorical columns.	2, 16, 1024	2	2	2	2	2	2	2	2	2	2	2
	Summary		Number of models built	37	31	33	54	54	39	29	50	39	30	47
			Best model ID	full_model_33	pVIF_model_8	rVIF_model_8	RFE_all_f_model_33	RFE_all_t_model_33	RFE_pvif_f_model_8	RFE_pvif_t_model_8	RFE_rvif_f_model_33	RFE_rvif_t_model_8	LASSO_min_model_8	LASSO_1se_model_33
			ROC AUC (training after 10-fold CV)	0.945	0.946	0.947	0.940	0.934	0.948	0.947	0.948	0.913	0.945	0.938
			ROC AUC (test)	0.936	0.933	0.930	0.926	0.917	0.936	0.935	0.937	0.662	0.926	0.926

Supplementary Table S6. Hyperparameter optimization in GBM. For each subset, the parameter values for the best models are determined by maximizing the ROC AUC. The highest AUC values for both the training and test sets are highlighted in green, while the best subset is circled in red.

[Supplementary Table S7]

eXtreme Gradient Boosting results

Algorithm name	Argument name	Argument description	Parameter space	"All in" n = 27	"Permissive" VIF n = 23	"Restrictive" VIF n = 20	RFE "all in" set with rerank F n = 10	RFE "all in" set with rerank T n = 9	RFE "permissive" VIF set with rerank F n = 19	RFE "permissive" VIF set with rerank T n = 21	RFE "restrictive" VIF set with rerank F n = 17	RFE "restrictive" VIF set with rerank T n = 1	Lasso lambda.min n = 25	Lasso lambda.1se n = 14
XGBoost	*col_sample_rate*	The number of features randomly sampled as candidates at each split.	seq(0.1,0.5,0.1), seq(0.55, 1, 0.05)	0.85	0.85	0.7	0.7	0.7	0.7	0.7	0.7	0.7	1	0.7
	ntrees	The number of trees built.	5, 50, 100, 200, 500, 1000, 5000, 10000	1461	1466	460	998	1264	992	446	955	870	200	956
	max_depth	The number of splits each tree is allowed to make.	3, 5, 10, 20, 30	20	20	30	30	30	30	30	30	30	30	30
	col_sample_rate_per_tree	Relative change of the column sampling rate for each tree.	0.5, 0.9, 1	0.9	0.9	1	1	1	1	1	1	1	0.5	1
	sample_rate	Training data (rows subsampled) used to train each tree.	0.5, 0.632, 0.8, 0.95, 1	0.8	0.8	0.5	0.5	0.5	0.5	0.5	0.5	0.5	0.5	0.5
	min_rows	Minimum number of observations for a leaf to split.	1, 5, 10, 20	5	5	1	1	1	1	1	1	1	1	1
	min_split_improvement	Minimum relative improvement in order for a split to happen.	0, 1e-8, 1e-6, 1e-4	0	0	0	0	0	0	0	0	0	0	0
	reg_lambda	L2 regularization term on weights.	seq(0,20,1)	17	17	0	0	0	0	0	0	0	9	0
	Summary		Number of models built	8	8	9	14	15	14	9	14	15	3	14
			Best model ID	full_model_3	pVIF_model_3	rVIF_model_4	RFE_all_f_model_4	RFE_all_t_model_4	RFE_pvif_f_model_4	RFE_pvif_t_model_4	RFE_rvif_f_model_4	RFE_rvif_t_model_4	LASSO_min_model_2	LASSO_1se_model_4
			ROC AUC (training after 10-fold CV)	0.950	0.950	0.949	0.944	0.935	0.952	0.953	0.952	0.914	0.941	0.943
			ROC AUC (test)	0.938	0.939	0.935	0.927	0.917	0.941	0.937	0.939	0.616	0.932	0.924

Supplementary Table S7. Hyperparameter optimization in XGBoost. For each subset, the parameter values for the best models are determined by maximizing the ROC AUC. The highest AUC values for both the training and test sets are highlighted in green, while the best subset is circled in red.

Supplementary Table S8

Selected variables	min	1st quartile	median	mean	3rd quartile	max	skewness	st.dev.
Tave01	-6.80	-2.98	-1.05	-1.62	-0.10	1.90	-0.73	2.31
Tave02	-3.70	-0.30	-0.10	0.85	2.30	6.10	0.42	2.06
Tave03	-1.00	3.90	5.30	5.35	6.80	10.80	-0.13	2.03
Tave04	1.70	9.10	10.20	10.31	11.50	16.30	-0.19	2.02
Tave05	8.50	13.18	14.55	14.25	15.22	19.60	-0.63	2.14
Tave06	12.70	17.70	18.70	18.87	19.70	22.90	-0.22	1.68
Tave07	11.80	20.60	22.90	21.93	23.50	24.90	-1.78	2.41
Tave08	11.50	19.35	20.80	20.73	22.10	25.10	-0.43	2.07
Tave09	9.90	16.07	17.35	17.40	19.10	21.30	-0.37	2.13
Tave10	2.90	9.80	11.00	11.29	13.20	15.60	-0.44	2.33
Tave11	-4.60	1.50	4.70	4.40	7.20	10.90	-0.29	3.70
Tave12	-2.90	-0.53	1.50	0.81	2.20	3.10	-0.73	1.66
Tave01 – Tave12	-6.80	5.50	10.30	11.87	19.40	25.10	0.14	7.47
Tmax01	-2.70	0.45	1.95	1.65	3.38	4.60	-0.49	2.02
Tmax02	-0.90	2.40	3.00	4.15	6.10	9.90	0.59	2.49
Tmax03	2.20	8.60	10.50	10.41	12.30	16.50	-0.37	2.56
Tmax04	6.10	14.90	15.80	16.12	17.60	23.40	-0.12	2.33
Tmax05	12.70	18.70	20.55	20.01	21.32	26.20	-0.79	2.60
Tmax06	17.00	23.30	24.40	24.63	25.70	29.40	-0.16	2.09
Tmax07	16.70	26.80	29.10	28.21	30.00	32.00	-1.66	2.83
Tmax08	16.80	25.30	27.20	27.05	28.70	32.30	-0.38	2.43
Tmax09	15.30	21.10	23.75	23.56	26.00	28.70	-0.32	2.85
Tmax10	7.50	15.20	16.40	16.84	19.30	21.60	-0.38	2.79
Tmax11	-0.80	5.80	7.80	8.33	10.80	15.20	-0.12	3.73
Tmax12	0.20	2.10	3.95	3.43	4.73	5.70	-0.61	1.67
Tmax01 – Tmax12	-2.70	10.70	15.80	17.38	25.50	32.30	0.06	8.29
Tmin01	-11.00	-6.35	-4.70	-4.89	-3.15	-0.70	-0.75	2.71
Tmin02	-6.80	-3.30	-2.80	-2.46	-1.30	2.40	0.16	1.78
Tmin03	-4.20	-1.00	0.20	0.28	1.50	5.30	0.18	1.68
Tmin04	-2.70	3.30	4.60	4.50	5.50	9.80	-0.31	1.85
Tmin05	4.10	7.80	8.55	8.48	9.50	13.10	-0.41	1.76
Tmin06	8.30	12.30	13.10	13.12	13.80	16.50	-0.38	1.32
Tmin07	7.00	14.62	16.50	15.64	17.00	18.30	-1.82	2.06
Tmin08	6.30	13.40	14.30	14.42	15.60	18.60	-0.48	1.75
Tmin09	4.50	10.10	11.40	11.25	12.60	14.10	-0.57	1.65
Tmin10	-2.00	4.40	5.80	5.73	7.00	9.70	-0.52	2.04
Tmin11	-8.30	-2.40	1.35	0.49	3.50	7.00	-0.42	3.75
Tmin12	-6.10	-3.33	-1.20	-1.82	-0.40	0.90	-0.80	1.73
Tmin01 – Tmin12	-11.00	0.40	4.80	6.37	13.30	18.60	0.21	6.70
PPT01	5.00	18.00	38.00	35.44	53.75	68.00	-0.11	20.70
PPT02	10.00	17.00	33.00	30.61	36.00	103.00	1.91	15.27
PPT03	4.00	16.00	25.00	26.94	31.00	93.00	1.92	15.17
PPT04	4.00	20.00	36.00	35.54	46.00	90.00	0.59	17.83
PPT05	34.00	49.00	77.00	87.08	105.50	221.00	1.47	46.74
PPT06	31.00	53.00	75.00	83.94	115.00	193.00	0.70	36.74

PPT07	15.00	44.25	55.00	63.67	76.00	197.00	1.51	34.36
PPT08	4.00	31.00	47.00	47.86	59.50	145.00	0.71	23.00
PPT09	10.00	31.75	42.00	48.50	67.00	116.00	0.48	23.05
PPT10	7.00	23.00	34.00	37.39	48.00	124.00	1.39	20.74
PPT11	0.00	2.00	25.00	25.98	46.00	87.00	0.43	22.85
PPT12	9.00	17.75	31.50	35.69	53.25	79.00	0.26	19.67
PPT01 – PPT12	0.00	23.00	34.00	40.19	53.00	221.00	1.82	26.62
Tave wt	-7.20	-2.80	-0.70	-0.98	0.50	4.20	0.07	2.15
Tave sp	2.10	9.20	10.20	10.16	11.20	14.30	-0.69	1.45
Tave sm	11.00	19.30	20.40	20.52	22.40	24.30	-1.03	2.09
Tave at	2.50	9.70	10.90	10.97	12.50	15.50	-0.57	2.11
Tmax wt	-4.10	0.10	1.80	2.01	3.58	7.50	0.26	2.34
Tmax sp	6.70	14.50	15.60	15.60	17.00	19.90	-0.49	1.77
Tmax sm	15.80	25.10	26.50	26.57	28.60	31.30	-0.97	2.43
Tmax at	7.20	14.10	16.20	16.04	17.80	21.00	-0.39	2.47
Tmin wt	-10.30	-5.60	-3.70	-3.97	-2.30	0.90	-0.06	2.06
Tmin sp	-2.40	4.00	4.80	4.73	5.40	8.70	-0.91	1.25
Tmin sm	6.10	13.50	14.20	14.47	16.00	17.70	-1.05	1.81
Tmin at	-2.20	4.80	5.85	5.91	7.38	10.00	-0.85	1.91
PPT wt	46.00	89.00	99.00	108.60	126.00	226.00	1.28	35.78
PPT sp	79.00	130.00	156.00	161.80	195.00	339.00	0.43	44.14
PPT sm	97.00	156.00	202.00	201.70	231.00	441.00	0.91	57.84
PPT at	41.00	87.00	125.00	125.90	171.80	278.00	0.18	53.02
MAT	0.70	9.20	10.00	10.16	11.40	14.10	-0.84	1.71
MAP	367.00	562.00	630.00	640.70	701.00	1213.00	0.78	121.59
AHM	10.60	28.00	32.60	32.71	37.20	62.90	0.19	7.15

Supplementary Table S8: Descriptive statistics of selected climate variables for the period 2010-2020.

Supplementary Table S9

Variables	VIF values						
	Model 0 n = 26	Model 1 n = 25	Model 2 n = 24	Model 3 n = 23	Model 4 n = 22	Model 5 n = 21	Model 6 n = 20
AHM	15.124	15.010	excluded	excluded	excluded	excluded	excluded
aspect_E	1.029	1.029	1.027	1.024	1.024	1.024	1.024
aspect_N	1.030	1.030	1.029	1.032	1.032	1.032	1.032
cropland	1.905	1.905	1.872	1.899	1.846	1.833	1.830
elevation	7.384	7.218	7.246	7.280	excluded	excluded	excluded
forest_cover1	7.725	7.707	7.710	1.413	1.416	1.417	1.414
forest_cover2	9.180	9.152	9.156	1.236	1.236	1.236	1.231
forest_cover3	11.828	11.778	11.779	excluded	excluded	excluded	excluded
forest_cover4	reference	reference	reference	reference	reference	reference	reference
MAP	10.813	10.710	2.091	2.079	2.050	2.048	2.031
MAT	6.170	6.167	4.603	4.635	2.189	2.188	2.159
NDVI_A	4.931	4.933	4.912	5.048	5.020	5.017	1.478
NDVI_T	5.080	5.057	5.042	5.168	5.127	5.121	excluded
pasture	1.285	1.277	1.277	1.257	1.212	1.189	1.188
population	1.120	1.118	1.117	1.117	1.115	1.115	1.117
PPT	3.272	2.131	2.134	2.122	2.121	2.118	2.127
PPT_month	2.921	2.214	2.217	2.194	2.187	1.883	1.887
railway	1.269	1.261	1.263	1.268	1.198	1.188	1.188
road	1.169	1.169	1.171	1.162	1.160	1.160	1.159
settlement	1.193	1.191	1.190	1.189	1.169	1.169	1.170
slope	3.227	3.219	3.195	3.184	2.232	2.187	2.175
Tave	23.371	excluded	excluded	excluded	excluded	excluded	excluded
Tave_month	23.331	4.596	4.604	4.659	4.635	2.633	2.589
Trange_month	14.694	6.991	7.017	7.055	6.686	excluded	excluded
Trange_seasonal	15.714	3.762	3.769	3.767	3.702	1.912	1.911
unemployment	1.259	1.256	1.228	1.120	1.113	1.113	1.113
water	1.076	1.076	1.076	1.071	1.067	1.067	1.067
ROC AUC (test)	0.859	0.859	0.859	0.839	0.839	0.839	0.840

Supplementary Table S9. Summary of VIF values across different iterations of logistic regression models.

Supplementary Figures S1

Serbia

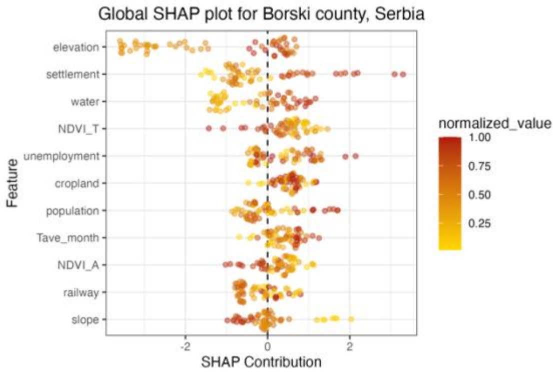

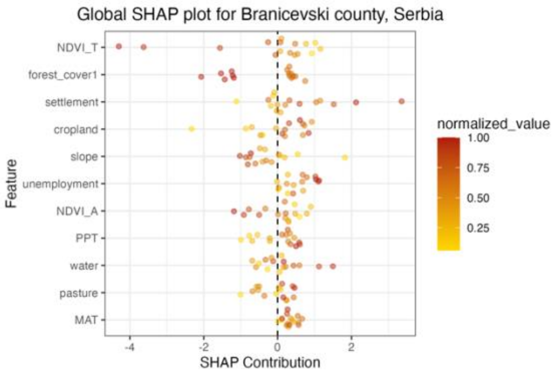

Romania

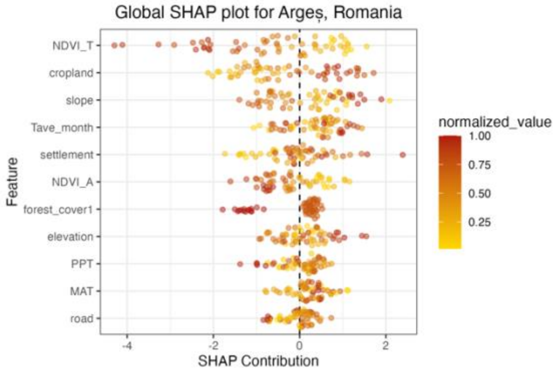

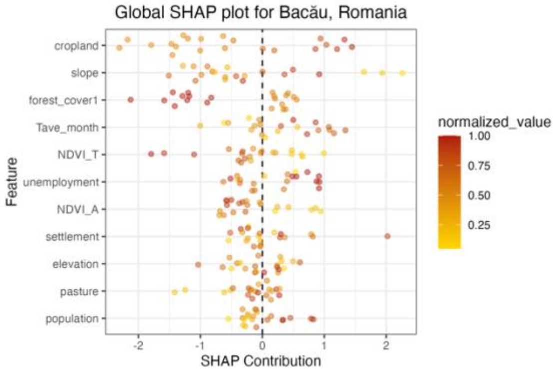

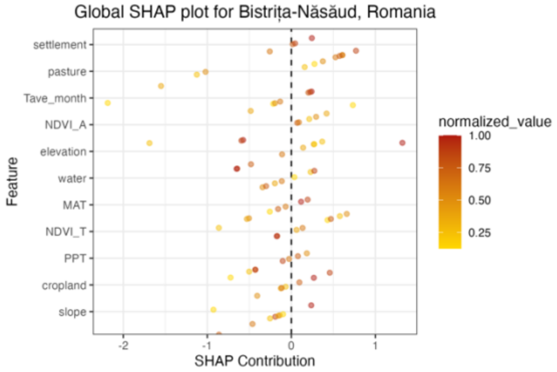

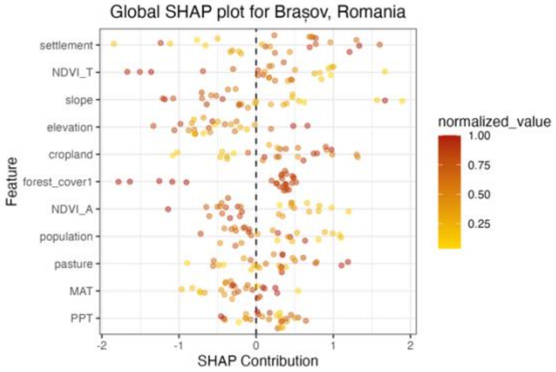

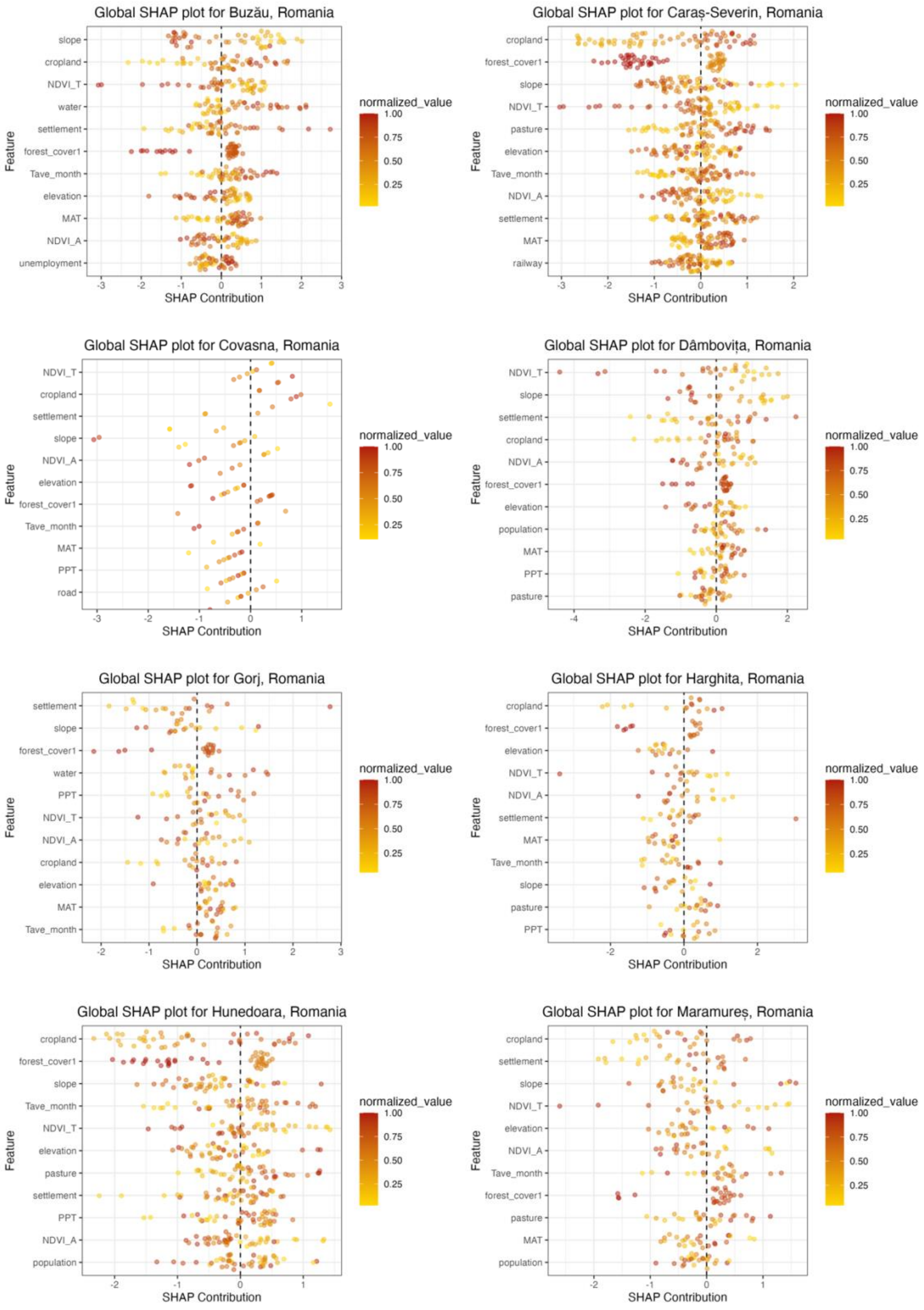
Global SHAP plot for Buzău, Romania
slope
cropland
NDVI_T
water
settlement
forest_cover1
Tave_month
elevation
MAT
NDVI_A
unemployment
Feature
SHAP Contribution
normalized_value
Global SHAP plot for Caraș-Severin, Romania
cropland
forest_cover1
slope
NDVI_T
pasture
elevation
Tave_month
NDVI_A
settlement
MAT
railway
Global SHAP plot for Covasna, Romania
NDVI_T
cropland
settlement
slope
NDVI_A
elevation
forest_cover1
Tave_month
MAT
PPT
road
Global SHAP plot for Dâmbovița, Romania
NDVI_T
slope
settlement
cropland
NDVI_A
forest_cover1
elevation
population
MAT
PPT
pasture
Global SHAP plot for Gorj, Romania
settlement
slope
forest_cover1
water
PPT
NDVI_T
NDVI_A
cropland
elevation
MAT
Tave_month
Global SHAP plot for Harghita, Romania
cropland
forest_cover1
elevation
NDVI_T
NDVI_A
settlement
MAT
Tave_month
slope
pasture
PPT
Global SHAP plot for Hunedoara, Romania
cropland
forest_cover1
slope
Tave_month
NDVI_T
elevation
pasture
settlement
PPT
NDVI_A
population
Global SHAP plot for Maramureș, Romania
cropland
settlement
slope
NDVI_T
elevation
NDVI_A
Tave_month
forest_cover1
pasture
MAT
population

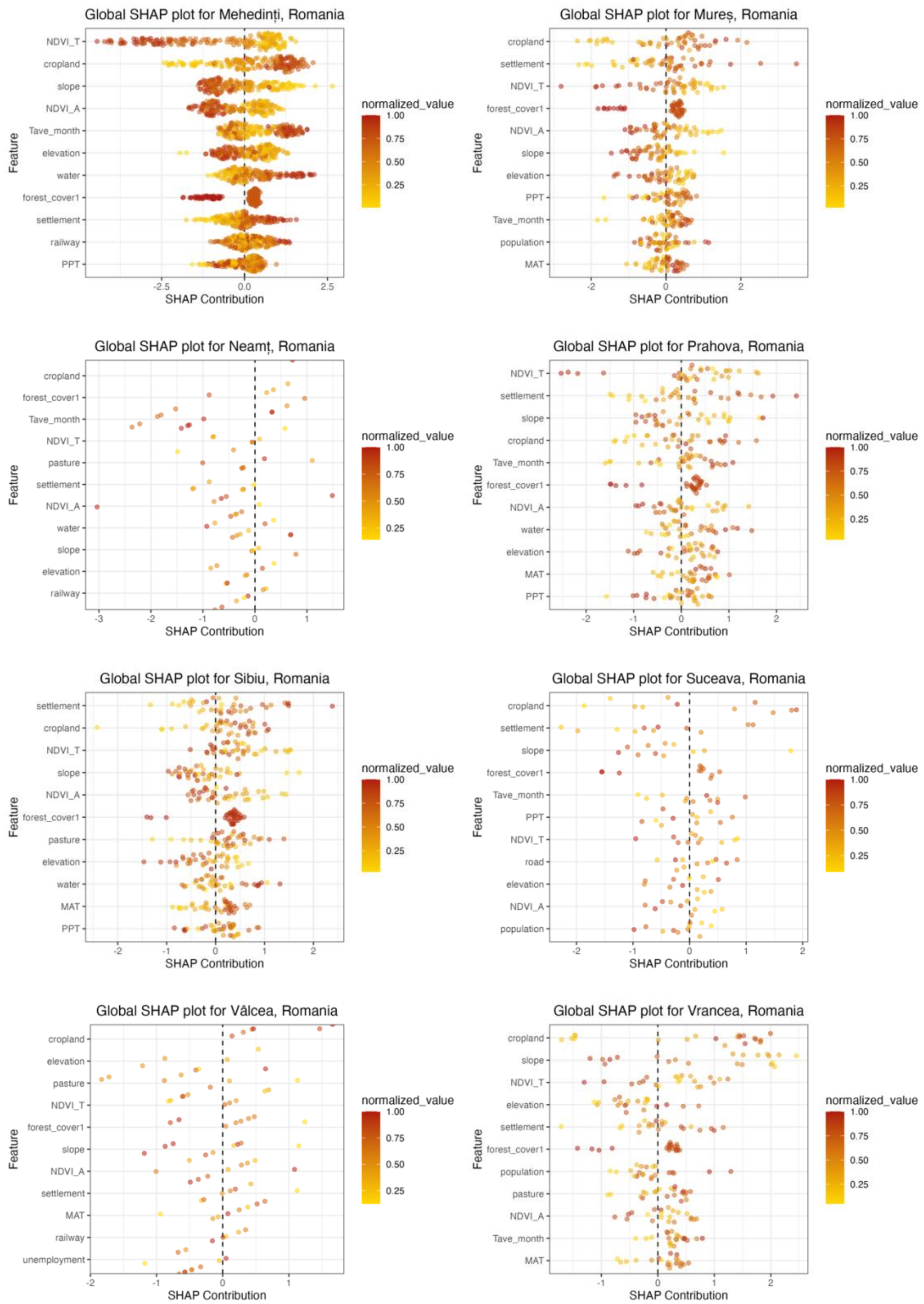
Global SHAP plot for Mehedinți, Romania
NDVI_T
cropland
slope
NDVI_A
Tave_month
elevation
water
forest_cover1
settlement
railway
PPT
-2.5
0.0
2.5
SHAP Contribution
Feature
normalized_value
1.00
0.75
0.50
0.25
Global SHAP plot for Mureș, Romania
cropland
settlement
NDVI_T
forest_cover1
NDVI_A
slope
elevation
PPT
Tave_month
population
MAT
-2
0
2
SHAP Contribution
Feature
normalized_value
1.00
0.75
0.50
0.25
Global SHAP plot for Neamț, Romania
cropland
forest_cover1
Tave_month
NDVI_T
pasture
settlement
NDVI_A
water
slope
elevation
railway
-3
-2
-1
0
1
SHAP Contribution
Feature
normalized_value
1.00
0.75
0.50
0.25
Global SHAP plot for Prahova, Romania
NDVI_T
settlement
slope
cropland
Tave_month
forest_cover1
NDVI_A
water
elevation
MAT
PPT
-2
-1
0
1
2
SHAP Contribution
Feature
normalized_value
1.00
0.75
0.50
0.25
Global SHAP plot for Sibiu, Romania
settlement
cropland
NDVI_T
slope
NDVI_A
forest_cover1
pasture
elevation
water
MAT
PPT
-2
-1
0
1
2
SHAP Contribution
Feature
normalized_value
1.00
0.75
0.50
0.25
Global SHAP plot for Suceava, Romania
cropland
settlement
slope
forest_cover1
Tave_month
PPT
NDVI_T
road
elevation
NDVI_A
population
-2
-1
0
1
2
SHAP Contribution
Feature
normalized_value
1.00
0.75
0.50
0.25
Global SHAP plot for Vâlcea, Romania
cropland
elevation
pasture
NDVI_T
forest_cover1
slope
NDVI_A
settlement
MAT
railway
unemployment
-2
-1
0
1
SHAP Contribution
Feature
normalized_value
1.00
0.75
0.50
0.25
Global SHAP plot for Vrancea, Romania
cropland
slope
NDVI_T
elevation
settlement
forest_cover1
population
pasture
NDVI_A
Tave_month
MAT
-1
0
1
2
SHAP Contribution
Feature
normalized_value
1.00
0.75
0.50
0.25

Hungary

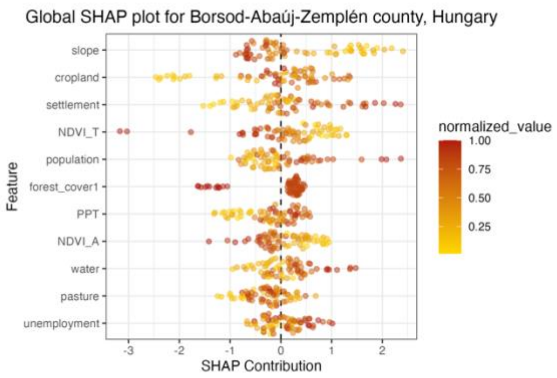

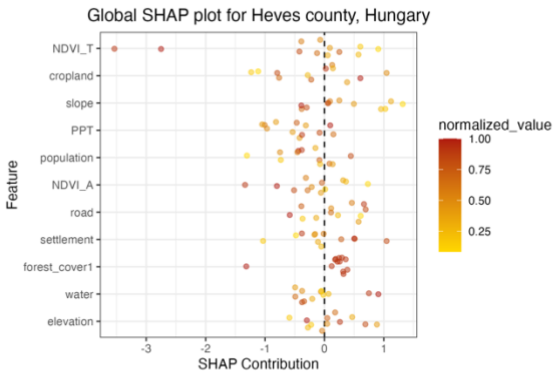

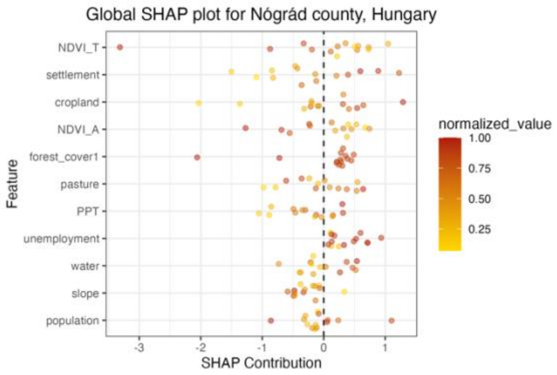

Poland

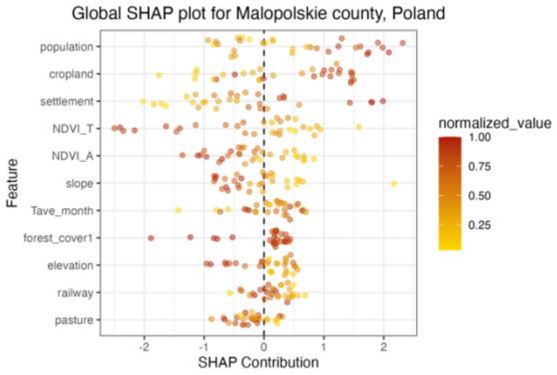

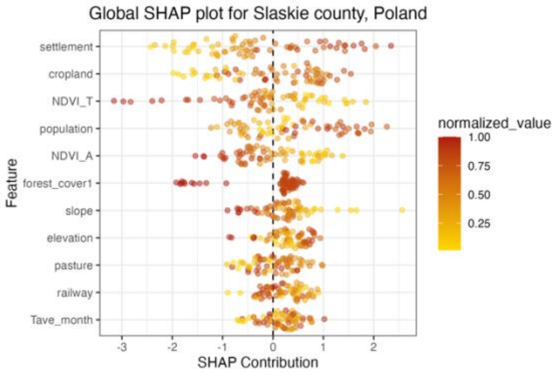

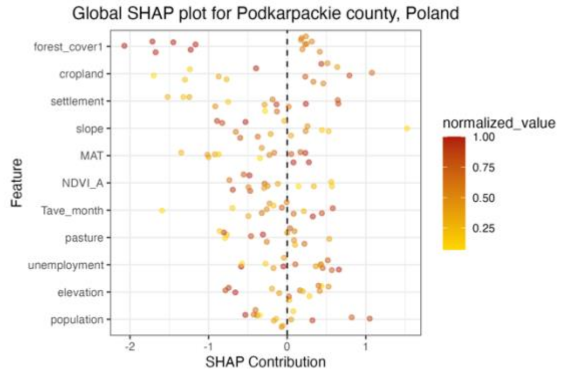

Ukraine

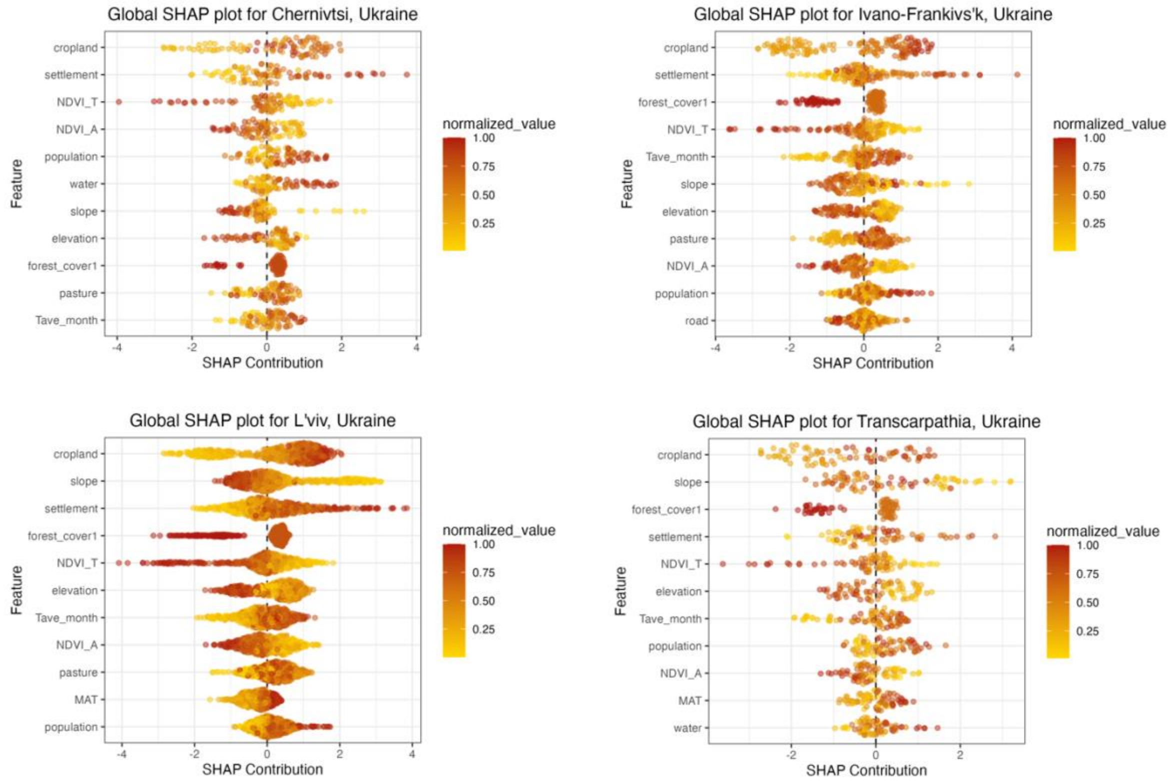

Supplementary Figures S1. Global SHAP Summary Plots for each county of the Carpathian region, using the XGBoost-RFE-pVIF-F model, showing the top 11 variables. Czech and Slovakian counties are merged into their respective countries due to the negative control generation process (see **Materials** section).

Supplementary Figure S2

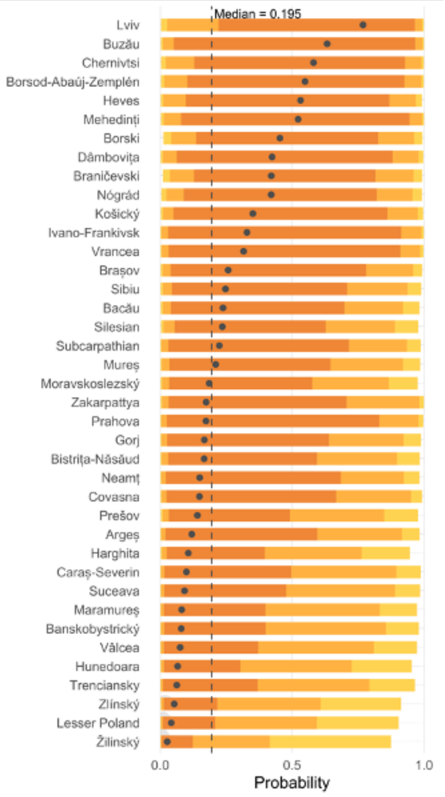

Supplementary Figure S2. Ridge plot showing the distribution of fire probabilities by county, arranged in descending order of median fire probability. The dots mark the individual median fire probabilities for each county. The 0.5, 0.8, and 0.95 quantile intervals are also shown, indicating the range where the respective percentages of fire probabilities fall.

Supplementary Figures S3

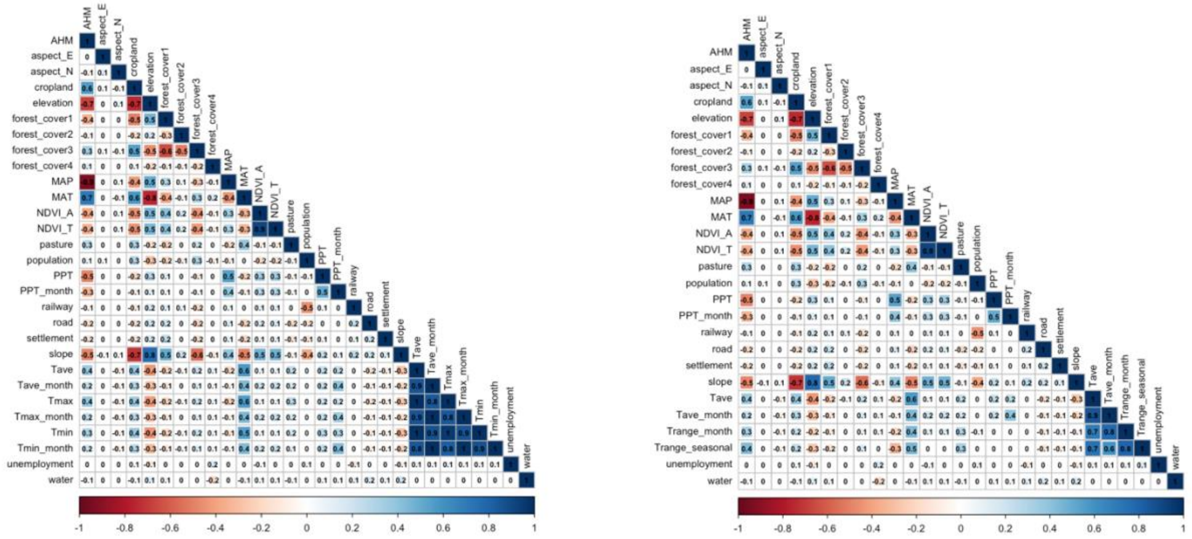

Supplementary Figures S3. Visualization of correlation matrices before (left panel) and after (right panel) data manipulation showing pairwise Spearman's correlations of our selected variables.

Supplementary Figures S4

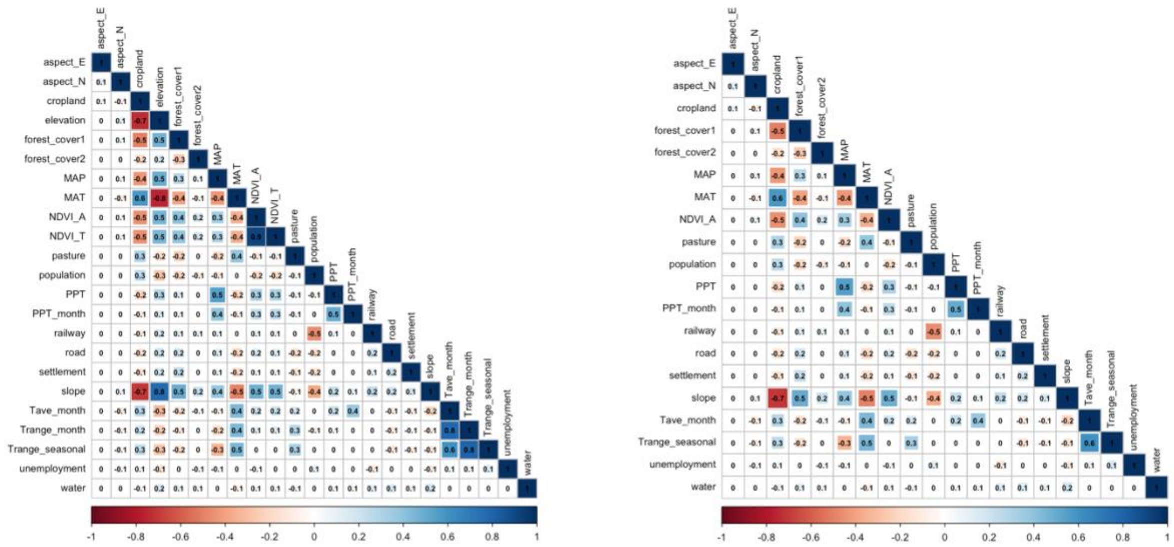

Supplementary Figures S4. Visualization of correlation matrices for model 3 (for "permissive" VIF with values below 10, left) and model 6 (for "restrictive" VIF with values below 5, right) showing pairwise Spearman's correlations of our selected variables.

Supplementary Figure S5

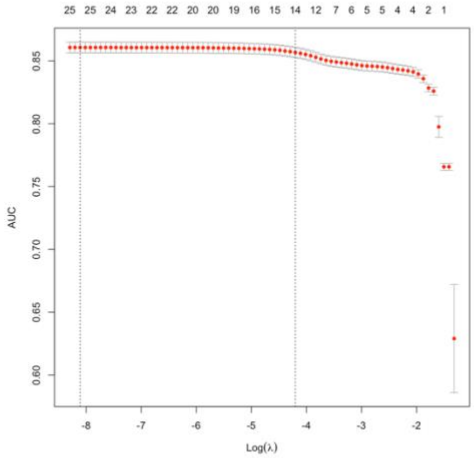

Supplementary Figure S5. Different λ values and the predictor subsets associated with those lambdas. $\lambda_{\min}$ (left vertical dashed line), λ_{1se} (right vertical dashed line).
